# NSUN2‐Mediated m^5^C Modification of *TGFB1* in Trophoblasts Remodels Macrophage Function to Prevent URSA

**DOI:** 10.1002/advs.77562

**Published:** 2026-09-13

**Authors:** Xiaoxiao Zhu, Xinkui Liu, Xiaoyan Yu, Qiaoqiao Han, Feifei Shi, Weiwei Song, Lu Liu, Chu Chu, Chenyang Shen, Zhen Zhang, Xia Li

**Affiliations:** ^1^ Innovative Institute of Chinese Medicine and Pharmacy Shandong University of Traditional Chinese Medicine Jinan China; ^2^ Key Laboratory of Traditional Chinese Medicine Classical Theory Ministry of Education Jinan Key Laboratory of Traditional Chinese Medicine Immunoregulation Traditional Chinese Medicine Immunoregulation Engineering Research Center of Shandong Province, Key Laboratory of Integrative Innovation in Traditional Chinese Medicine and Immunology of Shandong Province Shandong University of Traditional Chinese Medicine Jinan China; ^3^ Shanghai Institute of Immunology State Key Laboratory of Systems Medicine for Cancer Shanghai Jiao Tong University School of Medicine Shanghai China; ^4^ The Second Affiliated Hospital of Shandong University of Traditional Chinese Medicine Jinan China; ^5^ College of Traditional Chinese Medicine Shandong University of Traditional Chinese Medicine Jinan China

**Keywords:** 5‐Methylcytosine modification, macrophage, NSUN2, *TGFB1*, trophoblasts

## Abstract

Unexplained Recurrent Spontaneous Abortion (URSA) is a prevalent early pregnancy disorder with substantial implications for reproductive health, yet its pathogenesis remains poorly understood. Here, we uncover a previously unrecognized role of NOP2/Sun RNA methyltransferase 2 (NSUN2), an RNA 5‐methylcytosine (m^5^C) methyltransferase, in safeguarding pregnancy by remodeling trophoblast mediated macrophage function. We demonstrate that NSUN2 expression and global m^5^C modification levels are significantly diminished in villous tissues from URSA patients. Trophoblast‐specific ablation of *Nsun2* in mice resulted in embryonic absorption and decreased placental and fetal weight in mice underscoring its functional necessity in pregnancy maintenance. Mechanistically, loss of *NSUN2* in trophoblasts impairs m^5^C deposition on *TGFB1* mRNA, reducing the recruitment of the m^5^C reader YBX1, which in turn destabilizes the transcript and diminishes TGF‐β1 secretion, thereby suppressing M2 macrophage polarization and skewing the balance toward a pro‐inflammatory M1 phenotype. Notably, restoration of NSUN2 via adenovirus‐mediated gene delivery effectively rescues TGF‐β1 expression, rectifies macrophage imbalance, and alleviates embryo resorption in URSA mouse models. Collectively, our findings establish NSUN2 as an essential epigenetic regulator governing maternal‐fetal immune tolerance through m^5^C‐dependent stabilization of *TGFB1* mRNA, and identify the NSUN2 /YBX1/TGFB1 axis as a potential diagnostic biomarker and therapeutic target for URSA.

## Graphical abstract

NSUN2 and m^5^C decline in URSA villous tissues. Trophoblast *Nsun2* ablation disrupts macrophage‐mediated maternal‐fetal tolerance and triggers embryo resorption. Mechanistically, NSUN2‐YBX1 axis stabilizes m^5^C‐modified *TGFB1* mRNA to maintain TGF‐β1 secretion and M2 polarization, and restoring this signaling rescues maternal‐fetal immune tolerance to treat URSA.

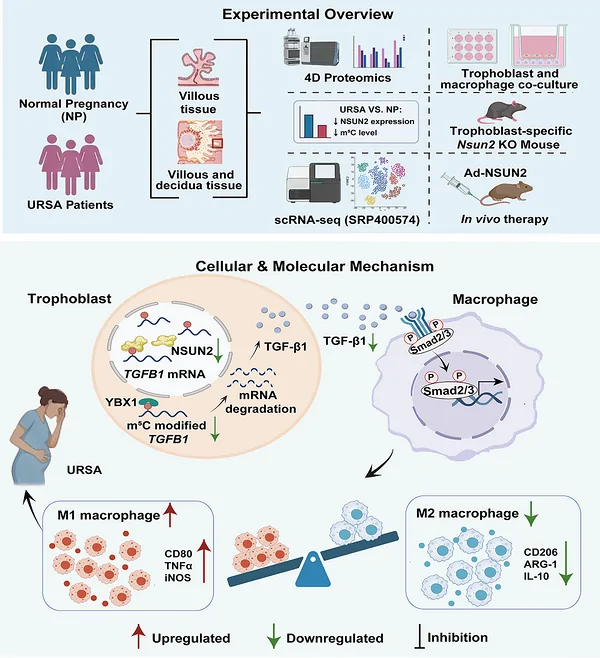

## 1 Introduction

Recurrent spontaneous abortion (RSA) represents one of the most profound challenges in reproductive medicine, affecting approximately 1%–5% of reproductive‐aged women worldwide and accounting for nearly 15% of all clinically recognized pregnancies [1, 2, 3]. Despite extensive clinical investigation, the underlying etiology remains elusive in approximately 50% of cases, which are consequently classified as unexplained recurrent spontaneous abortion (URSA) [4]. Beyond its direct reproductive health impact, URSA places profound psychological and emotional burdens on patients and substantial diagnostic and therapeutic challenges for clinicians [3, 5]. Elucidating the molecular underpinnings of URSA is therefore imperative for developing effective preventive and therapeutic strategies.

From the immunological perspective, successful pregnancy represents a remarkable paradigm of allogeneic transplantation, wherein the semi‐allogeneic fetus, carrying paternal antigens, must be protected from maternal immune surveillance throughout gestation [6, 7]. The immune state is orchestrated at the maternal‐fetal interface (MFI), where the placenta serves as both a physical barrier and an active immunological mediator [8, 9]. In particular, the villous trophoblasts function as critical immune educators, guiding the differentiation and activity of decidual immune populations via direct intercellular interactions and paracrine signaling, ultimately fostering maternal‐fetal immune tolerance [10]. Perturbation of this finely tuned trophoblast‐immune cell interplay can trigger the collapse of immune homeostasis, contributing to pregnancy complications including URSA [11, 12]. However, the molecular mechanisms by which trophoblasts regulate immune cells remain poorly defined.

Macrophages, as the one of the most abundant immune cell population at the MFI, exhibit remarkable phenotypic plasticity and play dual roles in pregnancy maintenance [13]. Decidual macrophages predominantly adopt an anti‐inflammatory M2 phenotype promoting tissue remodeling, angiogenesis, and immune tolerance [14]. Conversely, their shift toward a pro‐inflammatory M1 phenotype was related to the pathogenesis of URSA and other pregnancy disorders [15]. While accumulating evidence suggests that trophoblasts actively instruct macrophage polarization through paracrine signaling, the upstream molecular regulators governing this instructive capacity remain largely unexplored.

5‐Methylcytosine (m^5^C) is a prevalent post‐transcriptional RNA modification that regulates gene expression by influencing mRNA stability and translation efficiency [16]. Mechanistically, m^5^C exerts its regulatory functions through a coordinated “writer‐eraser‐reader” system. NOP2/Sun domain family (NSUN), the predominant mRNA m^5^C methyltransferase (writer) in mammals, catalyzes m^5^C deposition using S‐adenosyl‐L‐methionine as the methyl donor [17, 18]. Recent studies have further revealed that NSUN2, ALYREF and YBX1 can form a cooperative complex to regulate target mRNA stability in an m^5^C‐dependent manner [19]. The reader proteins ALYREF and YBX1 recognize m^5^C‐modified transcripts to facilitate nuclear export and prevent mRNA decay, respectively [18, 20]. Conversely, TET family proteins can demethylate m^5^C as “erasers”, enabling dynamic and reversible regulation [21]. This multilayered regulatory system allows m^5^C to influence diverse biological processes including cell differentiation and embryonic development [22, 23]. Beyond its physiological roles, NSUN2‐mediated m^5^C modification has been implicated in pathological conditions such as cancer, and inflammatory disorders [24, 25, 26]. Notably, emerging evidence has highlighted the critical role of m^5^C modification in reproductive physiology and gynecological diseases [27, 28]. However, whether NSUN2 contributes to the maintenance of maternal‐fetal immune tolerance, particularly through stabilizing trophoblast‐derived factors that regulate macrophage function, remains unexplored.

In this study, we found that NSUN2 expression and global m^5^C modification are significantly diminished in villous tissues from URSA patients. Through integrated transcriptomic, epitranscriptomic, and functional analyses, we uncover that NSUN2 maintains transforming growth factor‐beta 1 (TGF‐β1) expression by stabilizing its mRNA via m^5^C modification, thereby promoting the transformation of macrophages into M2 and sustaining immune tolerance at the MFI. Using trophoblast‐specific NSUN2 knockout mice and URSA models, we establish the functional necessity of this axis in pregnancy maintenance and demonstrate the therapeutic potential of NSUN2 restoration.

## 2 Results

### 2.1 NSUN2 and m^5^C Modification Levels are Downregulated in Villous Tissue of URSA Patients

**FIGURE 1:**
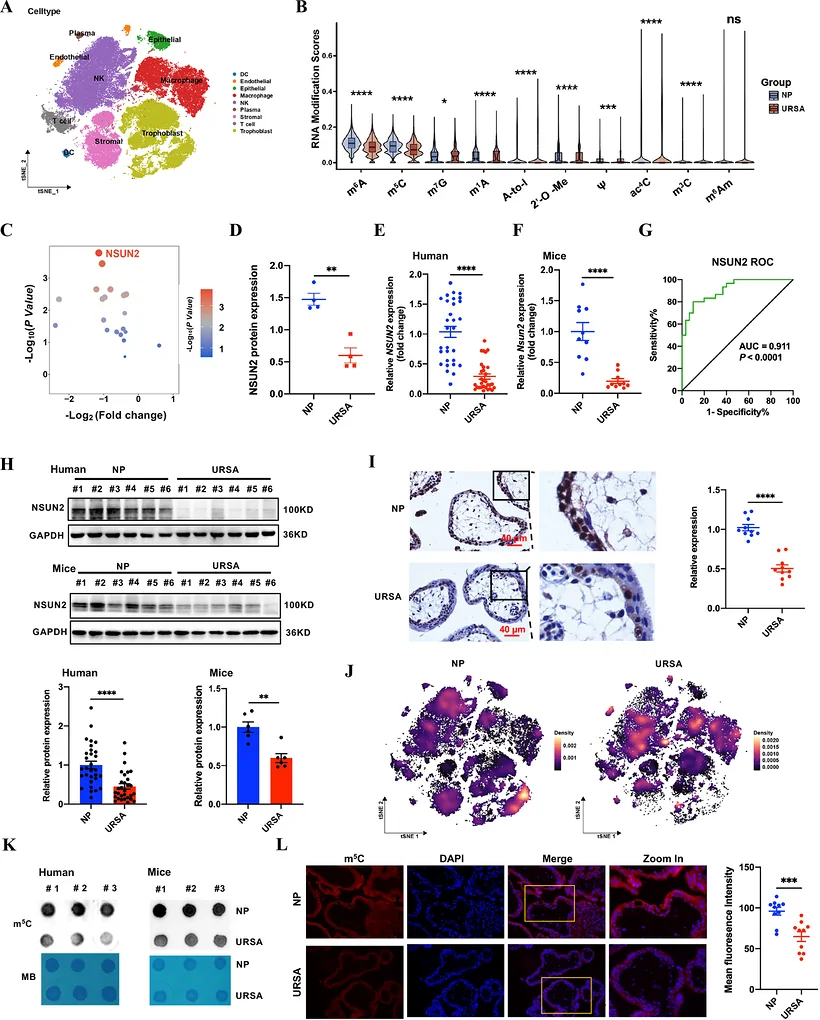
The expression of NSUN2 and m^5^C modification levels are downregulated in URSA. (A) tSNE projection of scRNA‐seq from human decidual and villi samples, colored by annotated cell type (nine distinct populations). (B) UCell RNA‐modification signature scores in NP and URSA cells. Signatures were scored only when at least two genes were detected. Groups were compared using two‐sided Wilcoxon rank‐sum tests; nominal *P* < 0.05 was considered significant. (C) Volcano plot displays m^6^A and m^5^C regulatory proteins of NP and URSA patient by the 4D proteome (*n* = 4). (D) NSUN2 proteins level in the 4D proteome (*n* = 4). (E, F) *NSUN2* mRNA levels in villous tissues (*n* = 30) and mice placental tissues (*n* = 10) from NP and URSA cases. (G) Assessing the diagnostic utility of *NSUN2* in villous tissues using ROC curve analysis (*n* = 30). (H) NSUN2 protein levels in villous tissues (*n* = 30) and mice placental tissues (*n* = 6) from NP and URSA cases. (I) IHC analysis NSUN2 expression in human villous tissues (*n* = 10). (J) tSNE plot showing the expression density of NSUN2 in MFI cells from URSA and NP groups. (K) Total m^5^C modification levels in human villous tissues and mouse placental tissues from NP and URSA groups (*n* = 3). (L) Representative IF images of m^5^C (red) and DAPI‐stained nuclei (blue) in human villous sections from NP and URSA patients. The zoom‐in views correspond to the areas highlighted by yellow boxes. Quantitative analysis of mean m^5^C fluorescence intensity in trophoblasts from NP and URSA groups (*n* = 10). Error bars represent means ± SEM. ns: no statistical difference, ^*^ *p* < 0.05, ^**^ *p*< 0.01, ^***^ *p* < 0.001, ^****^ *p* < 0.0001.

Numerous studies have demonstrated that RNA modifications play pivotal roles in the pathogenesis and progression of various diseases, including pregnancy‐related disorders [29, 30, 31]. In this study, the scores of multiple RNA modifications in trophoblast cells were evaluate using single‐cell sequencing (scRNA‐seq) data (SRP400574) of decidual and villous tissues from URSA patients and normal pregnancy (NP) controls. Unsupervised clustering identified nine distinct cell types (Figure 1A, Figure S1A). Notably, m^6^A and m^5^C modifications were the most abundant and were significantly reduced in trophoblast cells from URSA samples compared to NP controls (Figure 1B). We next analyzed the expression levels of all known m^6^A and m^5^C regulatory proteins in villous tissues from NP and URSA patients using quantitative proteomics analysis.

The 4D label‐free dataset identified 6766 proteins, of which 5054 had valid URSA/NP quantitative ratios. Principal‐component analysis of 3639 proteins quantified in all eight samples clearly separated URSA from NP specimens, and the mean within‐group Pearson correlation coefficients were 0.975 for NP and 0.935 for URSA, indicating satisfactory reproducibility (Figure S1B,C). Using fold change ≥ 1.5 or ≤ 0.67 and *P* < 0.05, 1,472 proteins were differentially expressed in URSA villous tissues, including 488 upregulated and 984 downregulated proteins. Analysis of RNA‐modification regulators revealed reduced abundance of the m^5^C‐associated proteins. Among quantified NSUN family members, NSUN4 and NSUN5 were unchanged, NSUN3/6/7 were undetectable, whereas NSUN2 was significantly downregulated in URSA villous tissues (Figure 1C,D, Figure S1D,E). This reduced expression of NSUN2 was further validated in an expanded cohort of URSA villous tissues and in placental tissues from a well‐established URSA mouse model (Figure 1E,F,H). ROC curve analysis identified NSUN2 expression reliably distinguishes URSA patients from normal controls, (AUC = 0.911, 95% CI: 0.842–0.980, Figure 1G). Immunohistochemical staining further confirmed diminished NSUN2 protein levels in villous sections from URSA patients compared to NP controls (Figure 1I).

To pinpoint the cellular origin of NSUN2 downregulation, we interrogated single‐cell transcriptomic profiles and found that trophoblasts exhibited the most pronounced decrease in NSUN2 expression in URSA samples (Figure 1J). Dot blot analysis further confirmed that m^5^C levels were concomitantly reduced in both human URSA villous tissues and placental tissues from URSA mice (Figure 1K). Given that NSUN2 was predominantly reduced in trophoblasts, we performed immunofluorescence staining on human villous sections to assess m^5^C levels specifically within trophoblasts. Quantitative analysis of immunofluorescence intensity verified that m^5^C modification was distinctly decreased in trophoblasts from URSA patients compared with NP controls (Figure 1L). Collectively, these findings establish that NSUN2 expression and m^5^C modification are significantly compromised in trophoblasts at the MFI in URSA, suggesting a potential role for NSUN2 in URSA disease pathogenesis.

### 2.2 Specific Ablation of NSUN2 in Trophoblasts Leads to Embryonic Resorption in Mice

**FIGURE 2:**
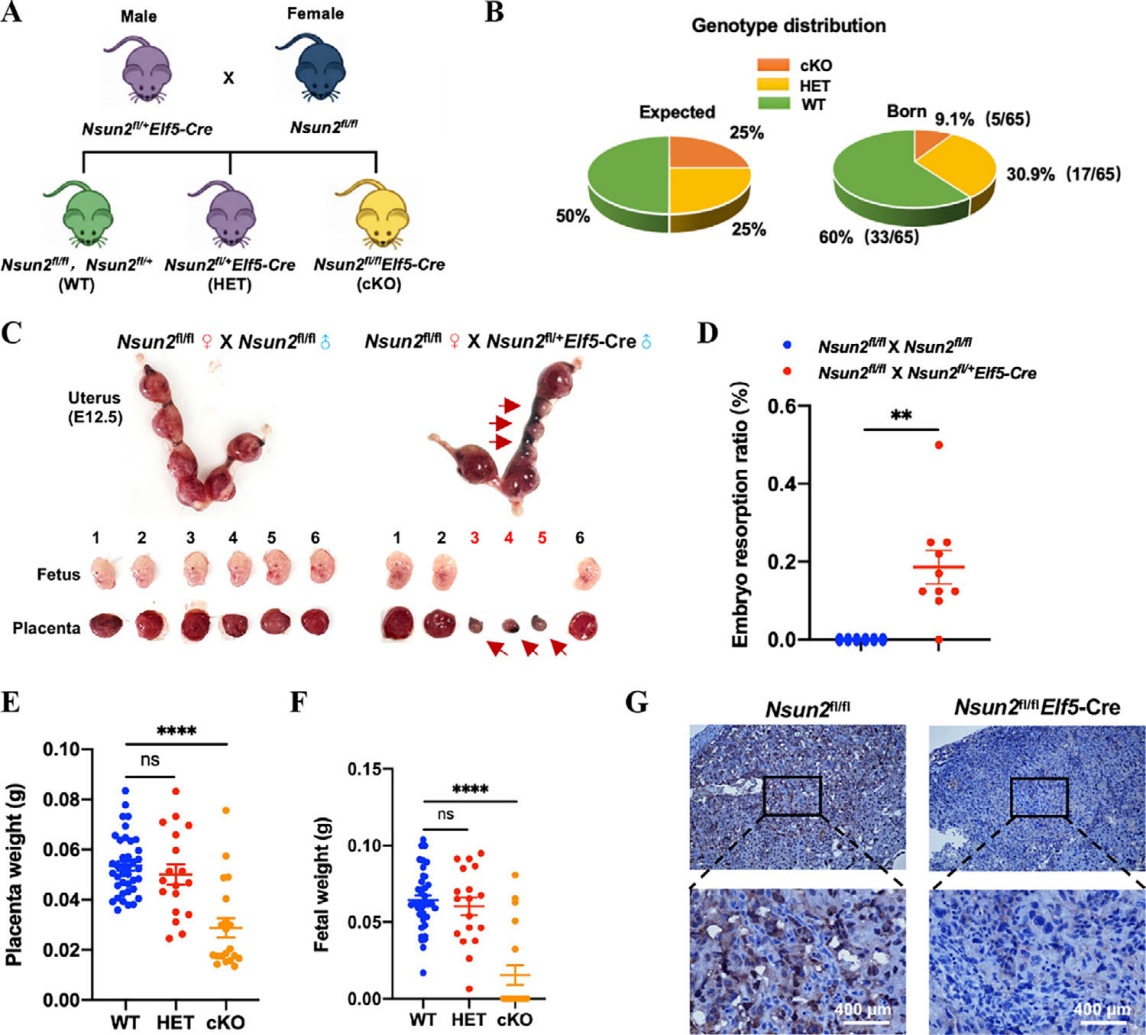
Loss of NSUN2 in trophoblasts leads to embryonic resorption in mice. (A) Mating strategy used to produce *Nsun2* cKO mice. (B) Genotype distribution of wild‐type (WT), heterozygous (HET), and cKO pups relative to the expected Mendelian inheritance ratio. The numbers of animals with specific genotypes over 65 pups were shown in the parentheses. (C) Representative uterine images of *Nsun2* ^fl/fl^ female mice mated with either *Nsun2* ^fl/fl^ or *Nsun*2^fl/+^ *Elf5‐*Cre males at E12.5. The red arrow indicates lethal embryos. (D) Embryo absorption rate of *Nsun2* ^fl/fl^ female mice mated with either *Nsun*2^fl/fl^ or *Nsun2* ^fl/+^ *Elf5‐*Cre males at E12.5 (*n* = 10). (E, F) Statistical analysis of placental weight and fetal weight in the WT, HET, and cKO group at E12.5 (*n* = 10). (G) IHC analysis the expression of NSUN2 in E12.5 *Nsun2* ^fl/fl^ and *Nsun2* ^fl/fl^ *Elf5‐*Cre mouse placental sections. Error bars represent means ± SEM. ns: no statistical difference, ^**^ *p* < 0.01, ^****^ *p* < 0.0001.

To directly investigate the functional requirement of NSUN2 in pregnancy maintenance, we generated a trophoblast‐specific conditional knockout mouse model by crossing *Nsun2*‐floxed (Nsun2^fl/fl^) mice with Elf5‐Cre transgenic mice (Figure 2A). Genetic analysis of offspring revealed that although heterozygous offspring were born at the expected Mendelian ratio of approximately 1:4 with no observable embryonic lethality or developmental abnormalities, only 9.1% of the expected conditional knockout (cKO) pups survived postnatally, a frequency substantially below the predicted Mendelian proportion (Figure 2B, Figure S1F). Morphological examination revealed clear signs of embryonic degradation and resorption in a subset of cKO fetus (Figure 2C, Figure S1H). Quantitative analysis demonstrated that *Nsun2* ^fl/fl^ females mated with *Nsun2* ^fl/+^ *Elf5‐*cre males exhibited significantly elevated embryo resorption rates compared with those mated with *Nsun2* ^fl/fl^ male mice (Figure 2D). Moreover, both placental and fetal weights were markedly reduced in *Nsun2* ^fl/fl^ *Elf5‐*cre mice, indicating intrauterine growth restriction and stillbirth risk among cKO animals (Figure 2E,F). Ablation of NSUN2 protein in cKO placentas was confirmed by immunohistochemistry and western blotting (Figure 2G, Figure S1G). These results establish that NSUN2 expression in trophoblasts is indispensable for successful pregnancy outcomes.

### 2.3 NSUN2 Deficiency in Trophoblasts Promotes Pro‐Inflammatory Activation of Macrophages

**FIGURE 3:**
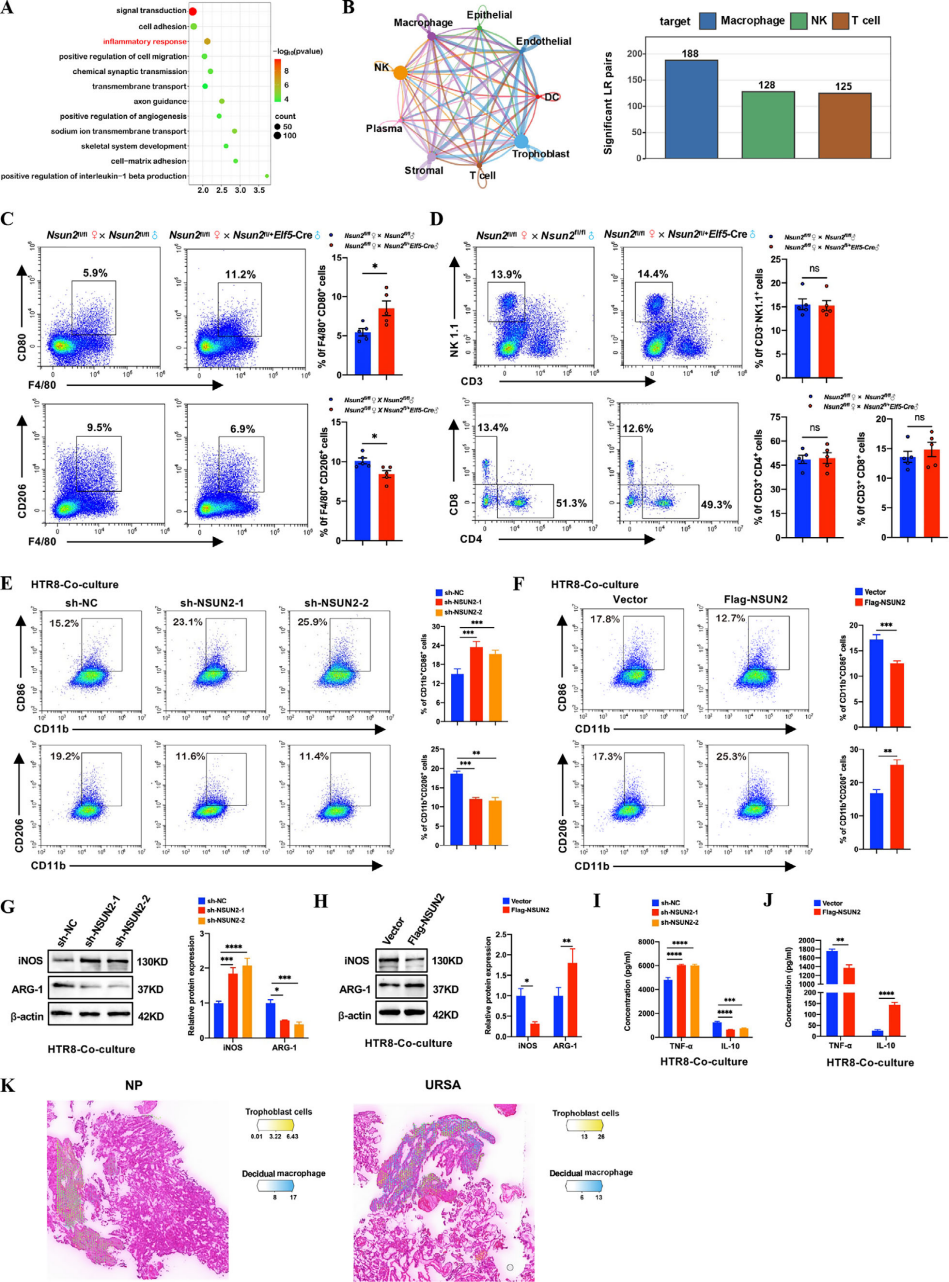
NSUN2 deficiency in trophoblasts drives the inflammatory activation of macrophages. (A) GO enrichment analysis was performed on the Biological Process (BP) ontology using differentially expressed genes (*P* < 0.05, |log2(fold change)| ≥ 1.0) by a hypergeometric test with Benjamini‐Hochberg correction (adjusted *P* < 0.05) and the top 15 terms with the smallest *P*‐values are shown. (B) CellChat summary of trophoblast‐directed immune‐cell communication. The circle plot shows inferred interaction counts, and the accompanying plot shows the number of significant ligand–receptor pairs from trophoblasts to macrophages, NK cells, and T cells in NP. (C, D) Flow cytometry analysis of the percentages of M1 cells (F4/80^+^ CD80^+^), M2 cells (F4/80^+^ CD206^+^), NK cells (CD3^−^NK1.1^+^), CD4^+^ T cell (CD3^+^ CD4^+^) and CD8^+^ T cell (CD3^+^ CD8^+^) from the decidual tissue of *Nsun2* ^fl/fl^ female mice mated with either *Nsun2* ^fl/fl^ or *Nsun2* ^fl/+^ *Elf5‐*Cre males at E12.5 (*n* = 5). (E, F) The proportion of M1 (CD11b^+^ CD86^+^) and M2 (CD11b^+^ CD206^+^) in THP‐1drived macrophages co‐cultured with HTR‐8. (G, H) WB showing the protein levels of iNOS and ARG‐1 in macrophages co‐cultured with HTR‐8. (I, J) ELISA analysis the level of TNF‐α and IL‐10 in the supernatant of co‐culture system with HTR‐8. (K) Spatial localization of macrophages and trophoblasts in NP and URSA of maternal‐fetal interface specimens, encompassing both decidual and chorionic villus tissues. (E–J) All data are representative of three independent experiments. Error bars represent means ± SEM. ns: no statistical difference, ^*^ *p* < 0.05, ^**^ *p* < 0.01, ^***^ *p* < 0.001, ^****^ *p* < 0.0001.

To explore the mechanisms underlying embryonic loss caused by NSUN2 depletion in trophoblasts, we established stable NSUN2‐knockdown and overexpression cell lines in HTR‐8 and JEG‐3 cells (Figure S2A–D). The endogenous biological functions of trophoblasts were subsequently assessed following NSUN2 modulation. The results showed that altering NSUN2 expression had no significant effect on proliferation (Figure S2E,F), apoptosis (Figure S2G,H), or migratory capacity (Figure S2I,J) in either cell line. Collectively, these findings suggest that NSUN2 deficiency does not cause embryonic resorption through direct effects on fundamental trophoblast behavior, implying that alternative mechanisms are responsible and warrant further investigation. So, RNA‐seq analysis was performed on NSUN2 depleted JEG‐3 cells, which revealed 938 downregulated and 430 upregulated genes compared with control cells (Figure S3A). GO analysis of differentially expressed genes showed a marked enrichment of the inflammatory response pathway, suggesting a potential role for NSUN2 in modulating immune‐related gene programs (Figure 3A). In light of the prominence of inflammatory pathways in the transcriptomic data, we next examined the cellular interactome at the MFI. Analysis of scRNA‐seq data (SRP400574) revealed that trophoblasts are predicted to interact with multiple immune cell types, including macrophages, NK cells, and T cells. Among these, trophoblast‐to‐macrophage communication exhibited the largest number of significant interactions (Figure 3B). Notably, NSUN2‐positive trophoblasts exhibited enhanced intercellular communication with macrophages compared with NSUN2‐negative trophoblasts, suggesting that NSUN2 expression level may influence trophoblast‐macrophage crosstalk (Figure S3B,C). These findings suggest that NSUN2 may influence pregnancy outcomes by modulating trophoblast‐macrophage crosstalk.

To determine whether this NSUN2‐associated crosstalk indeed affects macrophage status in vivo, we analyzed the immune cell composition in conditional *Nsun2* knockout mice. The results showed that *Nsun2* ^fl/fl^ females mated with *Nsun2* ^fl/+^ *Elf5‐*cre males exhibited a significant increase in the proportion of M1 macrophages and a concomitant decrease in M2 macrophages, whereas the percentages of NK cells, CD4^+^ T cells, and CD8^+^ T cells remained unchanged (Figure 3C,D, Figure S3D). Based on these findings, we speculate that trophoblast‐specific knockout of *Nsun2* may influence pregnancy outcomes by modulating macrophage function at the MFI. To further corroborate the direct regulatory effect of NSUN2 on macrophage polarization, we established an in vitro co‐culture system of trophoblasts with THP‐1‐derived macrophages and primary human decidual macrophages (Figure S3E). Flow cytometry analysis confirmed that co‐culture with NSUN2‐knockdown trophoblasts increased the proportion of CD11b^+^CD86^+^ M1 macrophages and decreased that of CD11b^+^CD206^+^ M2 macrophages, whereas NSUN2 overexpression produced the opposite effects in THP‐1‐derived macrophage(Figure 3E,F, Figure S3F,G).

In addition, co‐culture with NSUN2‐knockdown trophoblasts significantly upregulated the expression of M1 markers (CD86, NOS2, TNF‐α) and downregulated M2 markers (ARG‐1, IL‐10) at both the mRNA and protein levels, while NSUN2 overexpression elicited the opposite changes (Figure 3G–J, Figure S3H–M). Besides, co‐culture with primary human CD45^+^CD14^+^ decidual macrophages showed consistent results, that is, knockdown of NSUN2 in trophoblasts significantly upregulated M1 markers (NOS2, TNF‐α) and downregulated M2 markers (IL‐10, ARG‐1) at both transcriptional and translational levels, whereas NSUN2 overexpression reversed these effects (Figure S3N–Q). To further consolidate the preferential crosstalk between trophoblasts and macrophages in situ, we performed spatial transcriptomic analysis on maternal‐fetal interface specimens, encompassing both decidual and villus tissues. Notably, we observed substantial spatial co‐localization of trophoblasts and macrophages across these regions, providing direct morphological evidence supporting their intercellular communication at the MFI (Figure 3K). Taken together, these findings demonstrate that NSUN2 expression in trophoblasts critically regulates their ability to instruct macrophage polarization toward an immunoregulatory phenotype.

### 2.4 Knockdown of NSUN2 Significantly Suppresses TGF‐β1 Expression

**FIGURE 4:**
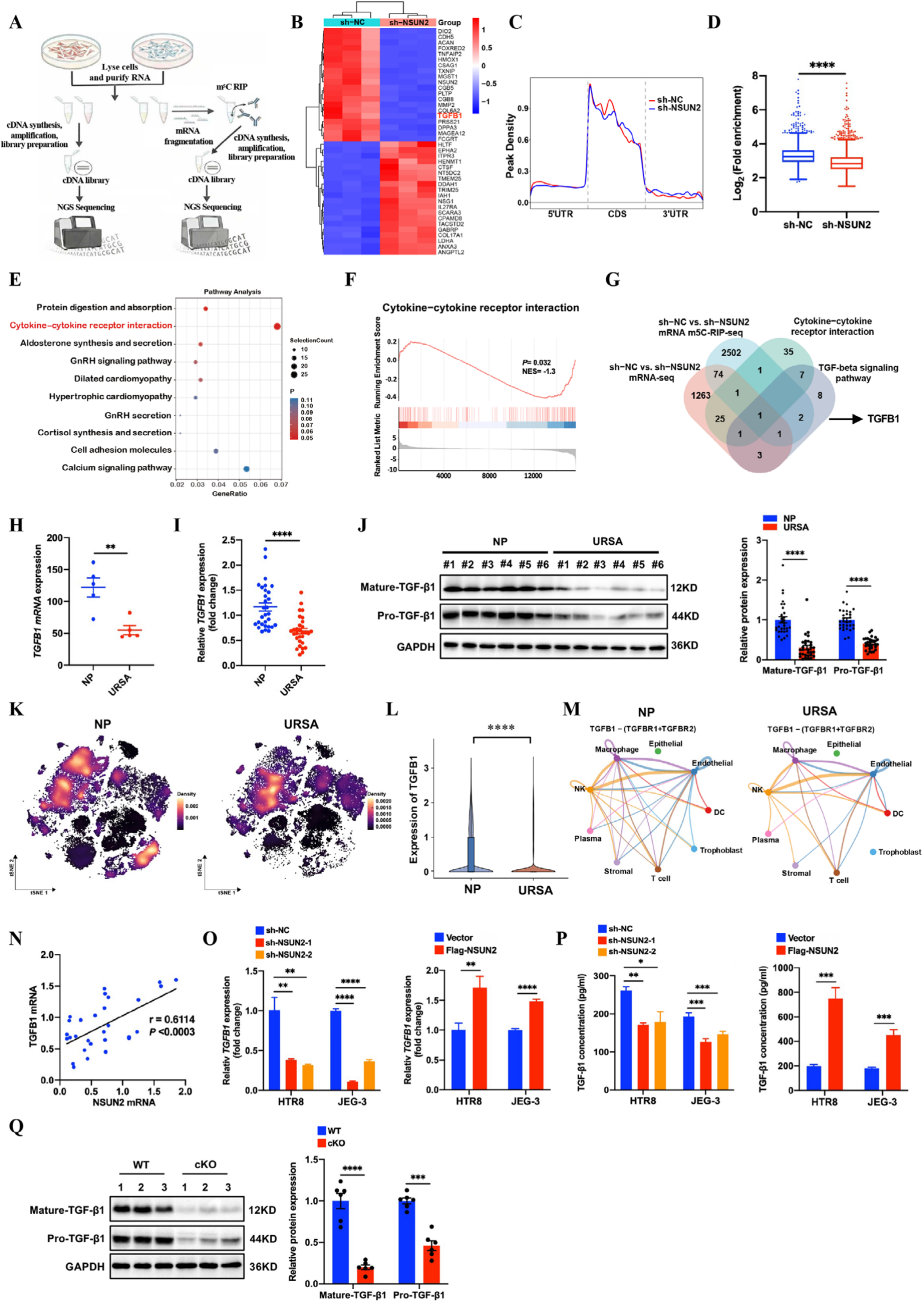
Knockdown of NSUN2 significantly suppresses TGF‐β1 expression. (A) Flow chart of the RNA‐seq and m^5^C MeRIP‐seq in control and NSUN2‐deficient JEG‐3 cells. (B) The heatmap displays the top 40 differently expression genes in RNA‐seq (*n* = 3). (C) Profiling the distribution and methylation levels of m^5^C sites in various mRNA segments (*n* = 3). (D) Box‐plot depicts the distribution of m^5^C peak enrichment levels of mRNAs in m^5^C MeRIP‐seq. (E) KEGG enrichment analysis of downregulated genes (*P* < 0.05, |log2(fold change)| ≥ 1.0) in NSUN2‐knockdown JEG‐3 cells. Enrichment was performed using a hypergeometric test with Benjamini‐Hochberg correction, and pathways with *P* < 0.05 were considered significantly enriched. The top 10 pathways ranked by Fold Enrichment are displayed. (F) GSEA showing enrichment of the cytokine‐cytokine receptor interaction pathway. (G) Venn diagram showing overlapping genes among the cytokine‐cytokine receptor pathway, TGF‐beta signaling pathway, different expression gene and differential m^5^C modified mRNA in the sh‐NSUN2 group. (H) The expression of *TGFB1* in villous tissues from NP and URSA patients based on RNA‐seq data (*n* = 5). (I, J) TGF‐β1 mRNA (*n* = 27) and protein levels (*n* = 30) in villous tissues of NP and URSA patients (*n* = 30). (K) t‐SNE visualization of normalized *TGFB1* expression in the integrated scRNA‐seq dataset. (L)) Comparison of normalized *TGFB1* expression in trophoblasts from NP and URSA samples; *P* values were calculated using a two‐sided Wilcoxon rank‐sum test. (M) CellChat‐inferred TGFB1‐receptor communication at the MFI in NP and URSA. (N) Correlation analysis between NSUN2 and TGFB1 expression (*n* = 30). (O) *TGFB1* mRNA levels in HTR8 and JEG‐3 NSUN2 knockdown and overexpression stable cell lines. (P) TGF‐β1 protein levels in supernatant of HTR8 and JEG‐3 NSUN2 knockdown and overexpression stable cell lines. (Q) TGF‐β1 protein levels in placental tissues from WT and cKO mice (*n* = 6). (O, P) All data are representative of three independent experiments. Error bars represent means ± SEM. ^*^ *p* < 0.05, ^**^ *p* < 0.01, ^***^ *p* < 0.001, ^****^ *p* < 0.0001.

As we have confirmed that NSUN2 in trophoblasts influences macrophage function, we proceeded to perform mRNA‐seq and m^5^C MeRIP‐seq in control and NSUN2‐deficient JEG‐3 cells to identify the downstream molecular mediators involved in this process (Figure 4A). The six UMI RNA‐seq libraries yielded 38.94–48.33 million clean reads per sample with Q30 values of 96.60%–96.90%; within‐group Pearson correlations based on log2(FPKM+1) were 0.9976–0.9982 in controls and 0.9957–0.9982 after NSUN2 depletion. The m^5^C MeRIP‐seq IP and input libraries yielded 32.71–44.84 million clean reads per library, with Q30 values of 96.89%–98.55% and mapping rates of 68.01%–74.65%. The complete RNA‐seq differential‐expression table and the complete m^5^C peak and differential‐peak tables are provided in the supporting information. RNA‐seq analysis identified extensive transcriptional changes upon NSUN2 depletion (Figure 4B). m^5^C MeRIP‐seq revealed that m^5^C sites were predominantly distributed across coding sequences, 5’ and 3’UTRs (Figure 4C), with global m^5^C enrichment significantly reduced in NSUN2‐deficient cells (Figure 4D). KEGG pathway analysis indicated that genes downregulated upon NSUN2 knockdown were prominently enriched in the cytokine‐cytokine receptor interaction pathway (Figure 4E), which was further corroborated by Gene Set Enrichment Analysis (GSEA) demonstrating significant inhibition of this pathway in sh‐NSUSN2 group (NES = −1.3, *P* = 0.032, Figure 4F). Additionally, ssGSEA revealed the activity of TGF‑beta signaling pathway was decreased in NSUN2‐depleted cells (Figure S4A,B).

Given the central role of TGF‐beta family members in immune regulation and macrophage polarization, we overlapped the genes from the cytokine‐cytokine receptor, TGF‐beta signaling pathways, with different expression genes and differentially m^5^C modified transcripts in the sh‐NSUN2 group. This analysis pinpointed *TGFB1* as a key candidate (Figure 4G). Moreover, independent RNA‐seq analysis of villous tissues revealed significant *TGFB1* downregulation in URSA patients compared with NP controls (Figure 4H). This reduction was further validated at both the mRNA and protein levels by qRT‐PCR and western blotting in human and mouse placental samples, as well as by ELISA‐based measurement of secreted TGF‐β1 in villous tissue homogenates (Figure 4I,J, Figure S4C–E). ROC curve analysis indicated that *TGFB1* expression effectively discriminated URSA from NP samples (AUC = 0.8477, *P* < 0.0001, Figure S4F). ScRNA‐seq analysis confirmed that *TGFB1* expression was significantly reduced specifically in trophoblast cells from URSA patients (Figure 4K,L), underscoring the pathological relevance of trophoblast‐derived TGF‐β1 in URSA.

Cell‐cell communication analysis further demonstrated that TGFB1‐TGFBR interactions between trophoblasts and macrophages were substantially weakened in URSA compared to NP conditions (Figure 4M, Figure S4G–J). This results suggesting that diminished trophoblast‐derived TGF‐β1 may compromise its paracrine signaling to macrophages at the MFI in URSA. Notably, *NSUN2* and *TGFB1* expression levels exhibited a significant positive correlation in human villous tissues (Figure 4N). Functional experiments confirmed that NSUN2 depletion in trophoblast cells markedly suppressed TGF‐β1 mRNA and protein expression, whereas NSUN2 overexpression significantly enhanced TGF‐β1 production (Figure 4O,P). Consistent with above findings, TGF‐β1 protein also showed a downward trend in mice placental tissues with trophoblast‐specific NSUN2 knockout (Figure 4Q). Overall, these results indicate that NSUN2 acts as a positive regulator of TGF‐β1 expression in trophoblast cells, and that disruption of this regulatory axis in URSA trophoblast cells may drive the polarization of decidual macrophages toward an M1 phenotype, thereby disrupting the immune tolerance microenvironment at the MFI.

### 2.5 NSUN2 Enhances the Stability of *TGFB1* mRNA Through m^5^C Modification

**FIGURE 5:**
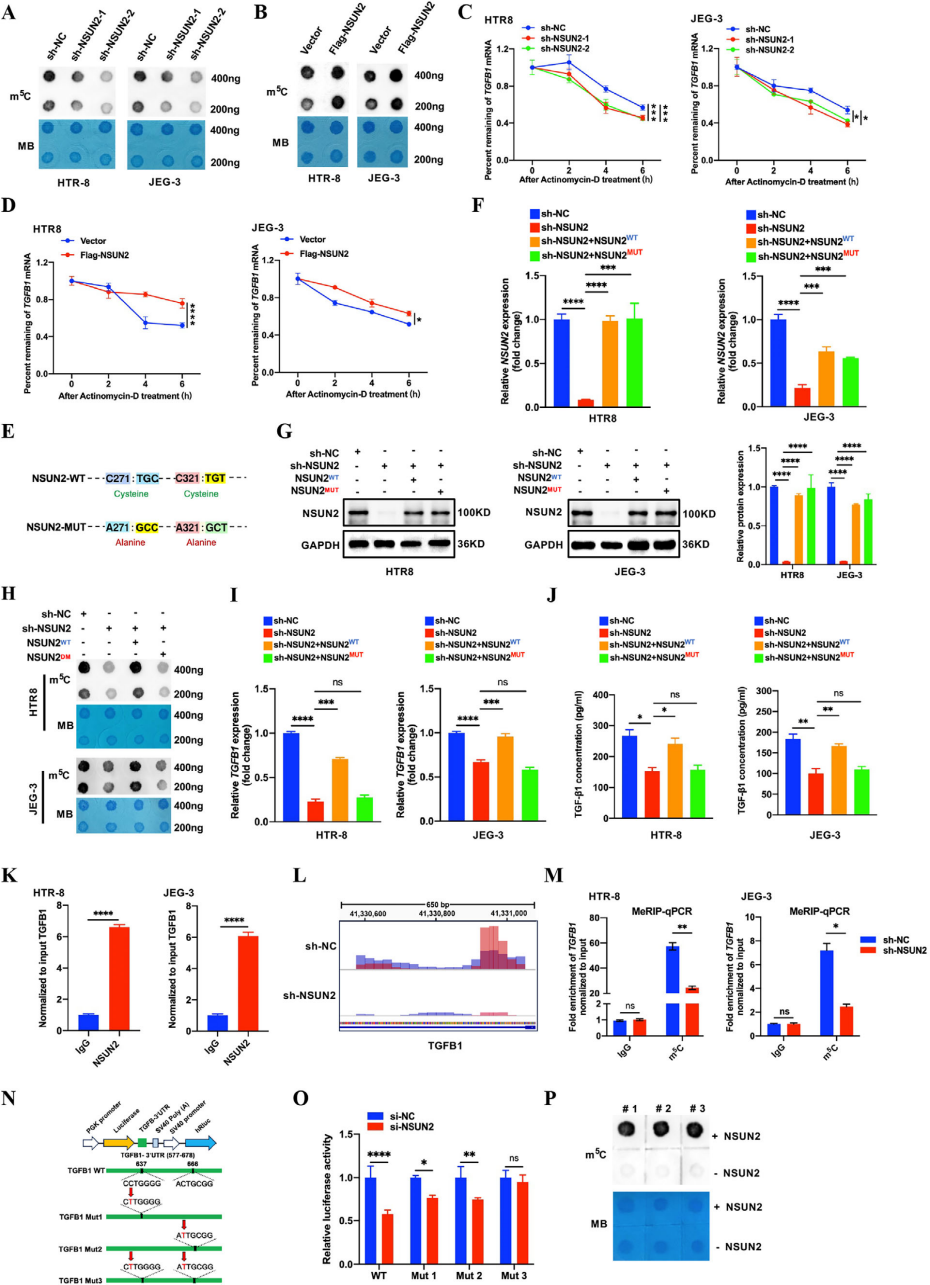
NSUN2 promotes the stability of *TGFB1* mRNA via m^5^C modification. (A, B) Total m^5^C modification levels in NSUN2‐knockdown and overexpressing HTR8/JEG‐3 stable cell lines. (C, D) Analysis of *TGFB1* mRNA half‐life in NSUN2‐knockdown and overexpressing HTR8/JEG‐3 stable cell lines. (E) Schematic of NSUN2 enzyme‐activity‐deficient mutants (C271A and C321A). (F, G) NSUN2 mRNA and protein levels in HTR8 and JEG‐3 NSUN2 knockdown stable cell line rescued with wild‐type (NSUN2‐WT) or mutant (NSUN2‐MUT) constructs. (H) Dot blot analysis of m^5^C modification levels. (I, J) qRT‐PCR and ELISA detection of TGF‐β1 mRNA and protein levels in HTR8 and JEG‐3 NSUN2 knockdown stable cell line rescued with NSUN2‐WT or NSUN2‐MUT plasmid. (K) RIP‐qPCR demonstrates NSUN2 binding to *TGFB1* mRNA. (L) Integrative Genomics Viewer (IGV) tracks show the distribution of m^5^C MeRIP‐seq reads across the *TGFB1* mRNA in control and NSUN2‐deficient JEG‐3 cells. (M) m^5^C MeRIP‐qPCR reveals the decrease in *TGFB1* mRNA m^5^C modification upon NSUN2 depletion. (N) *TGFB1* 3’UTR containing either wild type or mutant (C‐to‐T/A mutation) m^5^C sites was cloned into luciferase reporter vector. (O) Relative luciferase activity of the wild‐type and mutant form TGFB1‐3’UTR in HEK293T cells transfected with si‐NC or si‐NSUN2. (P) In vitro assay to assess NSUN2‐dependent m^5^C modification on *TGFB1* 3'UTR mRNA. All data in this figure are representative of three independent experiments. Error bars represent means ± SEM. ^*^ *p* < 0.05, ^**^ *p* < 0.01, ^***^ *p* < 0.001, ^****^ *p* < 0.0001.

We next investigated whether the methyltransferase activity of NSUN2 is required for its regulation of TGF‐β1 expression. Consistent with its role as a key m^5^C writer, NSUN2 knockdown globally reduced while NSUN2 overexpression increased total RNA m^5^C levels in trophoblast cells (Figure 5A,B). Actinomycin‐D chase assays revealed that NSUN2 knockdown significantly decreased *TGFB1* mRNA half‐life, whereas NSUN2 overexpression enhanced the transcript stability (Figure 5C,D), suggesting post‐transcriptional regulation. To determine whether NSUN2's effect on *TGFB1* requires its catalytic activity, we generated methyltransferase‐dead NSUN2 mutants (C271A and C321A) targeting critical cysteine residues essential for enzymatic function (Figure 5E). While both wild‐type (NSUN2‐WT) and mutant (NSUN2‐MUT) constructs restored NSUN2 mRNA and protein expression in NSUN2‐deficient cells (Figure 5F,G), only NSUN2‐WT rescued global m^5^C modification levels (Figure 5H). Critically, NSUN2‐WT but not NSUN2‐MUT restored TGF‐β1 expression at both mRNA and protein levels (Figure 5I,J), demonstrating that NSUN2's methyltransferase activity is required for TGF‐β1 up‐regulation. RIP‐qPCR assays confirmed direct association of NSUN2 with *TGFB1* mRNA, as evidenced by specific enrichment in NSUN2 immunoprecipitates compared to IgG controls (Figure 5K). M^5^C MeRIP‐seq data revealed prominent m^5^C enrichment within the 3'UTR of *TGFB1* mRNA, which was significantly diminished upon NSUN2 knockdown (Figure 5L). The reduction of m^5^C modification on *TGFB1* mRNA following NSUN2 depletion was further confirmed by m^5^C MeRIP‐qPCR (Figure 5M).

To evaluate the functional relevance of the TGFB1 3’UTR in NSUN2‐mediated regulation, we constructed wild‐type (WT) and mutant dual‐luciferase reporter plasmids, including two single‐site mutants (Mut1 and Mut2) and one double‐site mutant (Mut3), based on two previously reported NSUN2‐preferred m^5^C motifs (CNGCGG and CNGGG, where N denotes any nucleotide) found in the 3’UTR of *TGFB1* mRNA [17, 18] (Figure 5N). Dual‐luciferase reporter assays revealed that NSUN2 knockdown significantly reduced luciferase activity from the WT reporter, confirming the regulatory importance of this region. Notably, single site mutations resulted in only partial recovery of reporter activity, while the double mutation completely eliminated the regulatory influence of NSUN2 (Figure 5O), indicating both sites are critical for NSUN2‐dependent m^5^C modification and *TGFB1* mRNA stability. To further characterize direct m^5^C modification of *TGFB1* mRNA by NSUN2, we performed an in vitro methylation assay using purified recombinant NSUN2 protein and in vitro‐transcribed *TGFB1* 3’UTR mRNA, with S‐adenosylmethionine (SAM) as the methyl donor. Subsequent dot‐blot analysis with an anti‐m^5^C antibody showed that NSUN2 robustly enhanced the m^5^C signal on the 3’UTR mRNA, whereas no signal was detected without NSUN2 (Figure 5P). Collectively, these results establish that NSUN2 enhances *TGFB1* mRNA stability through m^5^C modification of its 3'UTR.

### 2.6 YBX1 Functions as the Reader to Recognize m^5^C‐Modified *TGFB1* mRNA and Maintain Its Stability

**FIGURE 6:**
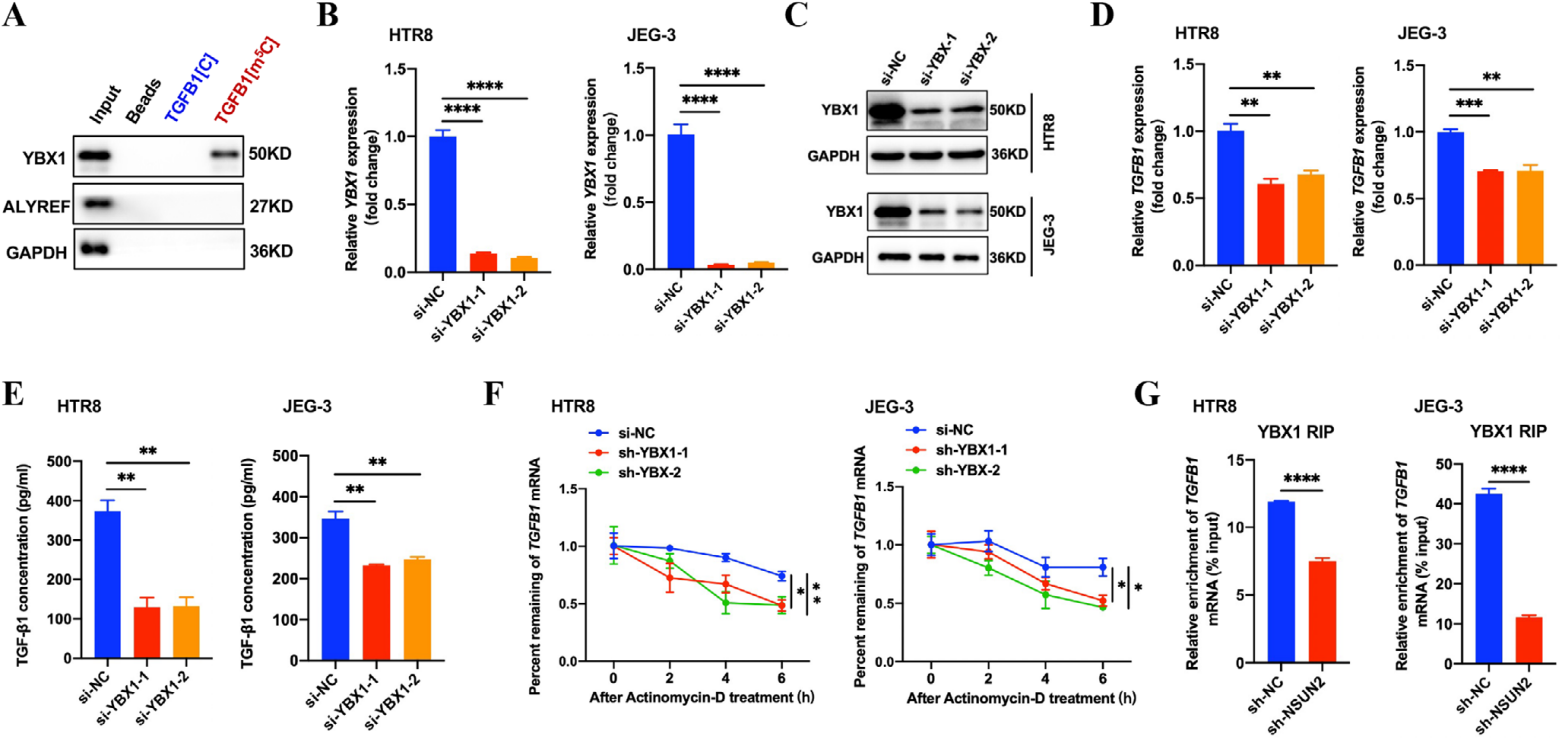
NSUN2‐m^5^C modification governs *TGFB1* mRNA stability via YBX1. (A) RNA‐protein pull‐down verified the specific binding between YBX1 and m^5^C‐modified *TGFB1* mRNA. (B, C) qRT‐PCR and WB confirmed the knockdown efficiency of YBX1 in HTR‐8 and JEG‐3 cells. (D, E) Relative *TGFB1* mRNA levels and secreted TGF‐β1 contents were detected after YBX1 silencing. (F) Actinomycin‐D chase assays assessed *TGFB1* mRNA half‐life upon YBX1 depletion. (G) RIP‐qPCR analysis showed that NSUN2 knockdown attenuated the interaction between YBX1 and TGFB1 mRNA. All data in this figure are representative of three independent experiments. Error bars represent means ± SEM. ns: no statistical difference, ^*^ *p* < 0.05, ^**^ *p* < 0.01, ^***^ *p* < 0.001, ^****^ *p* < 0.0001.

We next sought to identify the m^5^C‐binding protein responsible for regulating *TGFB1* mRNA stability. RNA pull‐down assays revealed that YBX1, but not ALYREF, specifically interacted with the m^5^C‐modified *TGFB1* 3′UTR (Figure 6A). To assess the functional significance of this interaction, we knocked down YBX1 using two independent siRNAs (Figure 6B,C), which led to marked reductions in both *TGFB1* mRNA abundance and secreted TGF‐β1 levels in HTR‐8 and JEG‐3 cells (Figure 6D,E). Actinomycin‐D chase assays further demonstrated that YBX1 depletion significantly shortened the half‐life of *TGFB1* mRNA, indicating that YBX1 functions to maintain *TGFB1* transcript stability (Figure 6F). Moreover, YBX1‐RIP‐qPCR confirmed that NSUN2 knockdown substantially weakened the association between YBX1 and *TGFB1* mRNA (Figure 6G). Collectively, these findings establish YBX1 as the functional m^5^C reader that recognizes m^5^C modified *TGFB1* mRNA and preserves its stability. In the context of NSUN2 deficiency, reduced m^5^C modification impairs YBX1 binding, thereby compromising mRNA stability and leading to subsequent decay.

### 2.7 Exogenous TGF‐β1 Rescues Macrophage Phenotype Induced by NSUN2 Deficiency in Trophoblasts

**FIGURE 7:**
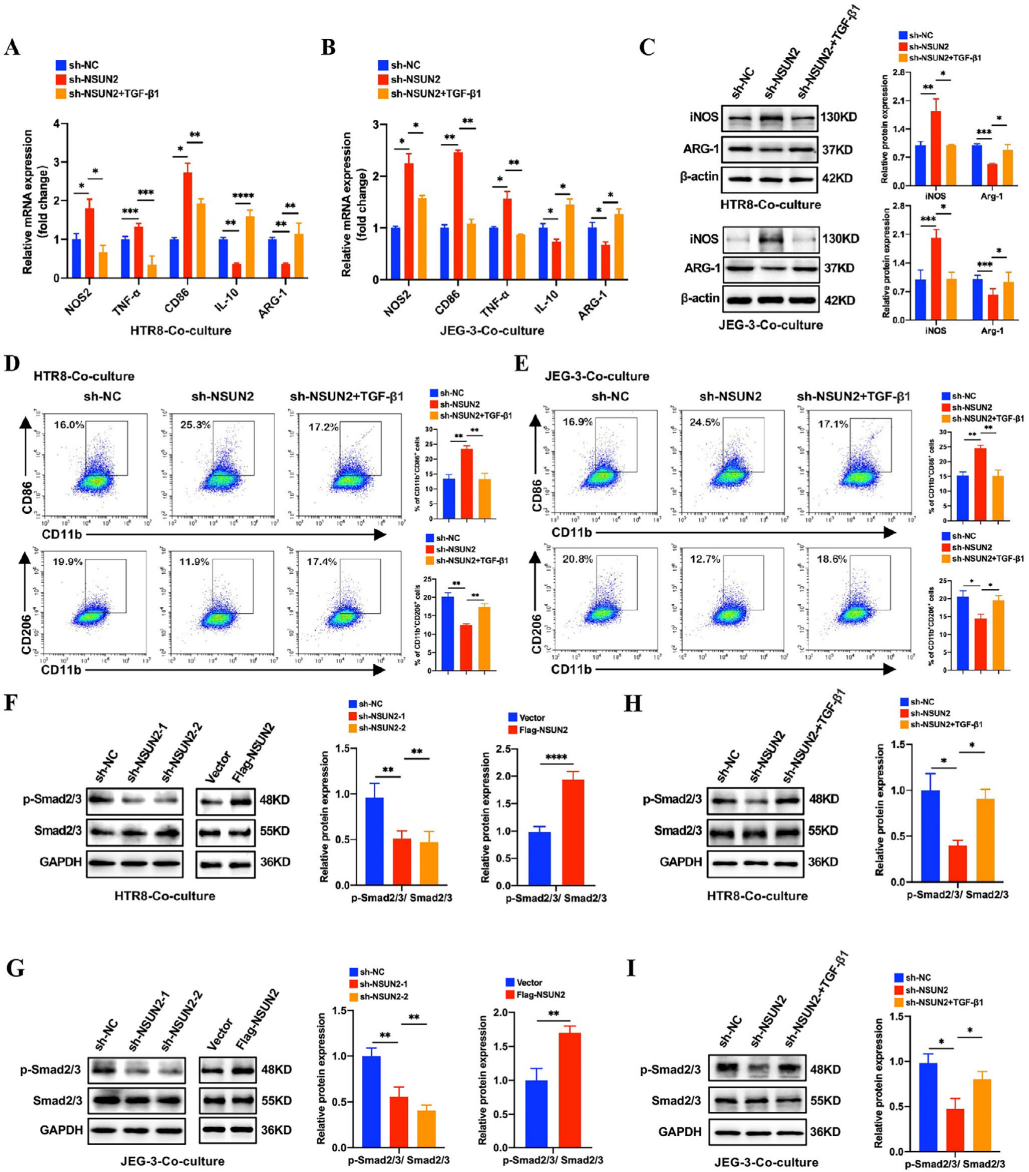
TGF‐β1 reverses NSUN2 knockdown‐induced macrophage inflammation through Smad2/3 activation. (A, B) *NOS2*, *CD86*, *TNF‐α*, *ARG‐1* and *IL‐10* mRNA level of macrophage in NSUN2‐knockdown co‐culture systems with or without TGF‐β1 supplementation. (C) iNOS and ARG‐1 protein levels in macrophages co‐cultured with NSUN2‐knockdown cells with or without TGF‐β1 supplementation. (D, E) The proportions of M1 (CD11b^+^ CD86^+^) and M2 (CD11b^+^ CD206^+^) macrophage in NSUN2‐knockdown co‐culture systems with or without TGF‐β1 supplementation. (F, G) Smad2/3 expression and its phosphorylation status in macrophages co‐cultured with NSUN2‐knockdown or NSUN2‐overexpressing trophoblasts. (H, I) Smad2/3 and p‐Smad2/3 protein levels in macrophages cocultured with NSUN2‐knockdown trophoblasts, with or without TGF‐β1 supplementation. All data in this figure are representative of three independent experiments. Error bars represent means ± SEM. ^*^ *p* < 0.05, ^**^ *p* < 0.01, ^***^ *p* < 0.001, ^****^ *p* < 0.0001.

Considering that TGF‐β1 plays a critical role in regulating macrophage polarization and maintaining immune balance at the MFI, we next investigated whether TGF‐β1 functionally mediates the effects of NSUN2 on macrophage polarization. Rescue experiments were therefore performed in the co‐culture system. Supplementation with recombinant human TGF‐β1 effectively reversed the M1 phenotype induced by NSUN2‐knockdown trophoblasts, as evidenced by reduced expression of M1 markers (CD86, TNF‐α, NOS2) and restored expression of M2 markers (IL‐10, ARG‐1) (Figure 7A–C). Flow cytometric analysis confirmed that TGF‐β1 supplementation normalized the proportions of CD11b^+^CD86^+^ M1 and CD11b^+^CD206^+^ M2 macrophages in co‐cultures with NSUN2‐deficient trophoblasts (Figure 7D,E). These results demonstrate that NSUN2 in trophoblasts primarily suppresses macrophage inflammatory activation through enhancing TGF‐β1 expression.

We next investigated the downstream signaling pathways in macrophages responsive to trophoblast‐derived TGF‐β1. Given the well‐established role of the Smad2/3 pathway in mediating TGF‐β1's immunomodulatory functions, we examined Smad2/3 phosphorylation in co‐cultured macrophages. NSUN2 knockdown in trophoblasts significantly reduced Smad2/3 phosphorylation in macrophages, whereas NSUN2 overexpression enhanced the phenotype (Figure 7F,G). Importantly, TGF‐β1 supplementation completely reversed the attenuation of Smad2/3 phosphorylation induced by NSUN2 deficiency (Figure 7H,I). These findings identify the Smad2/3 pathway as the downstream mediator through which trophoblast‐derived TGF‐β1 confers anti‐inflammatory effects on macrophages.

### 2.8 NSUN2 Rescues URSA In Vivo Through Macrophage Reprogramming

**FIGURE 8:**
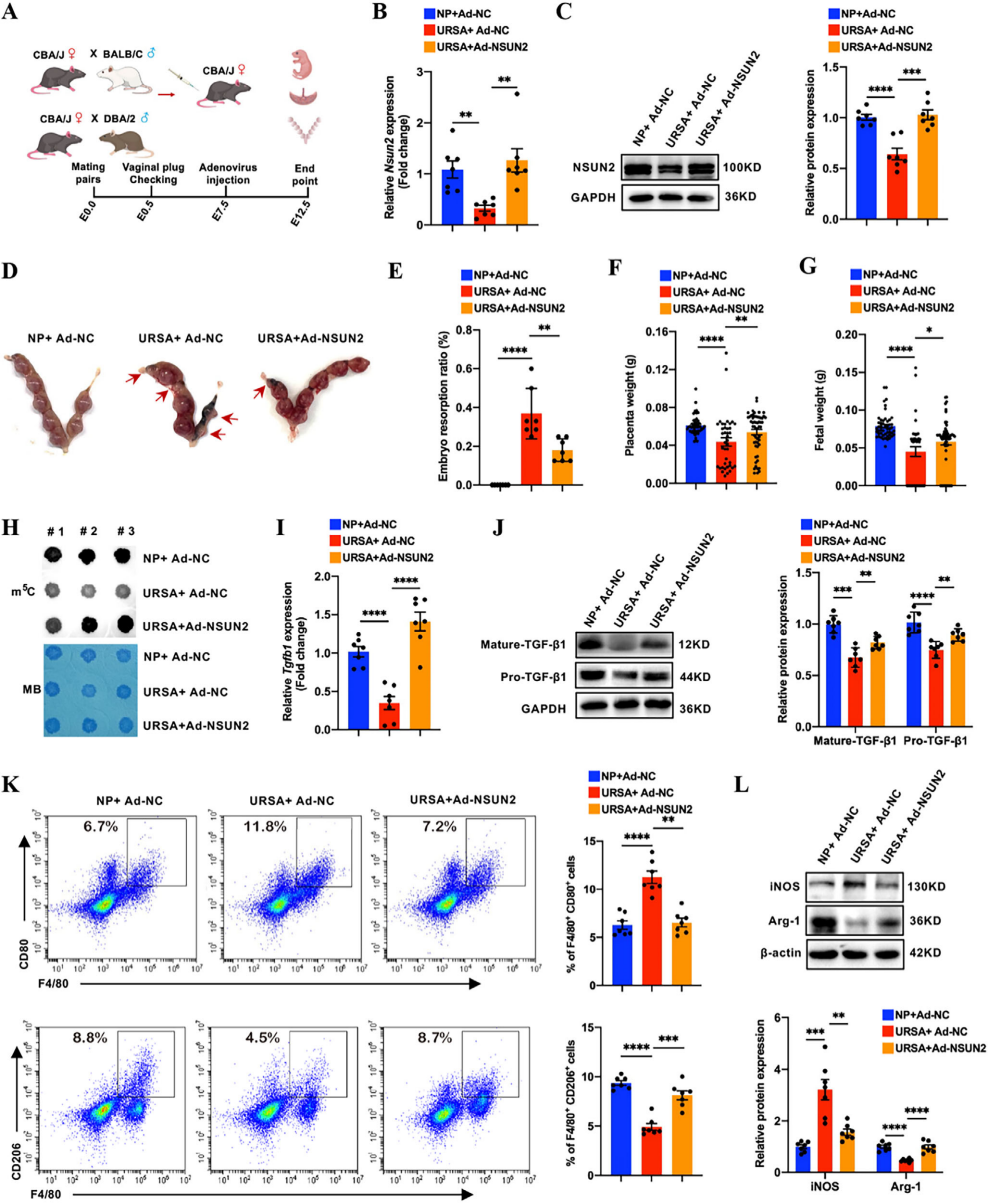
NSUN2 treatment alleviates embryo loss of URSA mice. (A) The flowchart shows the mouse processing procedure. URSA mice were injected with Ad‐NSUN2 via the tail vein at E7.5, and were euthanized at E12.5. (B, C) Assessment of NSUN2 mRNA and protein expression in mouse placental tissues (*n* = 7). (D) Representative uterine images of pregnant mice, arrows indicate the embryo resorption. (E) Embryo resorption rates of pregnant mice (*n* = 7). (F, G) Statistical analysis of placental weight and fetal weight of three group mice at E12.5 (*n* = 7). (H) Total m^5^C modification levels in placental tissues (*n* = 3). (I, J) Assessment of TGF‐β1 mRNA and protein expression in placental tissues (*n* = 7). (K) The proportions of M1 (F4/80^+^CD80^+^) and M2 (F4/80^+^CD206^+^) macrophage in decidual tissue (*n* = 7). (L) The protein levels of iNOS and ARG‐1in decidual tissue (*n* = 7). Error bars represent means ± SEM. ^*^ *p* < 0.05, ^**^ *p* < 0.01, ^***^ *p* < 0.001, ^****^ *p* < 0.0001.

To translate our mechanistic findings to an in vivo context and explore the therapeutic potential for NSUN2, we employed a well‐characterized URSA mouse model and administered NSUN2‐overexpressing adenovirus (Ad‐NSUN2) via tail vein injection at embryonic day 7.5 (E7.5) to achieve upregulation of NSUN2 in placenta (Figure 8A). Ad‐NSUN2 treatment successfully increased NSUN2 expression in placental tissues (Figure 8B,C). Strikingly, NSUN2 overexpression significantly reduced embryo resorption rates and increased both placental and fetal weights in URSA mice compared to Ad‐NC‐treated controls (Figure 8D–G). Dot blot analysis confirmed that Ad‐NSUN2 treatment elevated global m^5^C modification levels in URSA placental tissues (Figure 8H). Consistent with the in vitro findings, Ad‐NSUN2 administration dramatically increased TGF‐β1 expression in URSA placentas (Figure 8I,J). Examination of the decidual immune microenvironment revealed that he decidual of URSA mice is characterized by a marked elevation in M1 macrophages (F4/80^+^CD80^+^) and a concurrent reduction in M2 macrophages (F4/80^+^CD206^+^) (Figure 8K). Remarkably, Ad‐NSUN2 treatment effectively reversed this imbalance, restoring the M1/M2 ratio to the levels comparable to NP controls (Figure 8K). Western blotting analysis confirmed that Ad‐NSUN2 treatment downregulated the M1‐associated protein iNOS and upregulated the M2‐associated protein ARG‐1 in decidual tissues (Figure 8L). These results demonstrate that NSUN2 overexpression alleviates pregnancy loss in URSA mice by enhancing placental m^5^C modification and TGF‐β1 expression, thereby rectifying pathological macrophage polarization at the MFI.

## 3 Discussion

RSA is a common pregnancy complication that severely impairs the reproductive health of women of childbearing age [32]. Nearly half of RSA cases lack a clearly defined etiology, posing substantial challenges for clinical management [33]. Therefore, elucidating the underlying mechanisms is crucial for improving diagnostic and therapeutic strategies for RSA. This study demonstrates, for the first time, that the m^5^C methyltransferase NSUN2 acts as an essential immune regulator at MFI. We observed a marked reduction in NSUN2 expression and global m^5^C modification abundance in villous samples from URSA patients as well as in placental tissues from URSA mouse models. Trophoblast‐specific knockout of *Nsun2* in mice triggered elevated embryo resorption and a marked decrease in fetal and placental weights. Mechanistically, the lack of NSUN2 in the trophoblast inhibits TGF‐β1/Smad2/3 axis through m^5^C modification, thereby promoting polarization of M1 macrophages and ultimately driving URSA. Overall, our findings elucidate the pivotal role of the NSUN2‐TGF‐β1 axis in regulating the immune tolerance at MFI, thereby implicates targeting this axis as a potential therapeutic intervention for URSA.

RNA methylation, a widespread post‐transcriptional modification, controls diverse biological processes through its effects on RNA splicing, translation, transport, and stability, which has attracted substantial research interest [34, 35]. Evidence indicates that m^6^A methylation and its regulatory factors are dysregulated in pregnancy‐related diseases including RSA [30, 36], where they contribute significantly to disease mechanisms [37, 38]. Despite this, the functional significance of m^5^C, another major mRNA modification, in RSA development remains largely elusive. NSUN2, as the main enzyme catalyzing m^5^C formation, has been shown to be closely associated with various diseases. In tumors, high expression of NSUN2 promotes cell proliferation, migration and inhibits apoptosis by stabilizing *FABP5, ENO1, QSOX1*, which is associated with poor prognosis of osteosarcoma, colorectal cancer, and non‐small cell lung cancer [17, 39, 40]. In colitis NSUN2 attenuates colitis by destabilizing *IL17A/F* mRNA via m^5^C [26]. In preeclampsia, enoxaparin alleviates disease progression by enhancing trophoblast function via NSUN2‐mediated m^5^C methylation [28]. The integration of multi‐omics approaches, including proteomics, transcriptomics, and epitranscriptomics, has proven to be a powerful strategy for elucidating the molecular mechanisms of complex diseases [41]. In this study, analysis of 4D proteomics, scRNA‐seq, and an expanded URSA villous cohort revealed that NSUN2 expression and m^5^C levels were markedly decreased in URSA trophoblasts. ROC analysis further confirmed that NSUN2 expression reliably distinguishes URSA patients from normal controls, quantitatively validating the close association between reduced trophoblastic NSUN2 and URSA. More importantly, our in vivo animal experiments further elucidated the pathogenic mechanism of NSUN2 in URSA. Trophoblast‐specific *Nsun2* knockout in mice significantly increased embryo resorption rate and reduced fetal and placental weight. These findings strongly support that downregulation of NSUN2 expression and the consequent reduction of m^5^C methylation in trophoblasts are key molecular events driving URSA, highlighting the critical mechanistic significance of the NSUN2/m^5^C axis in the pathogenesis of URSA.

Successful pregnancy depends on the establishment and maintenance of immune tolerance microenvironment at MFI. An altered M1/M2 macrophage balance is recognized as an important contributor to URSA [6]. In this study, NSUN2 knockdown in trophoblasts enriched inflammatory response genes, and cell‐cell communication analysis revealed close trophoblast‐macrophage interaction. Functional experiments confirmed that trophoblast‐specific *Nsun2* knockout increased M1 and decreased M2 macrophages in decidual tissues, while in vitro knockdown promoted M1 polarization and suppressed M2 differentiation, indicating that NSUN2 maintains immune balance by mediating trophoblast‐macrophage communication. Notably, this effect appears largely macrophage‐selective, as we observed no significant changes in decidual NK, CD4^+^, or CD8^+^ T cell proportions in *Nsun2* cKO mice, suggesting NSUN2 deficiency does not broadly disrupt all immune lineages. We acknowledge, however, that we did not assess functional markers or effector molecule expression of these cells, and therefore we cannot formally exclude the possibility of functional changes that might affect the microenvironment. Nevertheless, the absence of quantitative changes in these immune populations, together with our observed macrophage‐related phenotypes, supports our focus on macrophage‐mediated regulation.

The TGF‐β family, a group of pleiotropic cytokines, acts as a central modulator in cell differentiation, apoptosis, and tissue homeostasis [42]. Among the TGF‐β superfamily proteins, TGF‐β1 is the most widely expressed and functionally diverse subtype [43]. As an executor of immune tolerance and an anti‐inflammatory factor, TGF‐β1 is involved in various physiological processes such as embryonic development, tissue homeostasis, injury repair [44]. During early pregnancy, TGF‐β1, predominantly secreted by trophoblast cells, not only regulates trophoblast invasion and migration to promote spiral artery remodeling, but also modulates decidual immune cell function to establish the immune tolerance microenvironment [45, 46, 47]. TGF‐β1 is beneficial for the establishment of immune tolerance microenvironment by promoting the conversion of naïve CD4^+^ T cells into Tregs, suppressing pro‐inflammatory cytokine release from macrophages, and promoting macrophages polarization to an M2 phenotype [43, 47]. Disturbances in TGF‐β1 levels and the TGF‐β1 signaling pathway are directly associated with the occurrence of pregnancy related diseases [48, 49].

Our study demonstrated that NSUN2 knockdown led to significant negative enrichment in both the cytokine‐cytokine receptor interaction and TGF‐bata signaling pathways. As a key molecule shared by these two pathways, the level of TGF‐β1 in the villus tissue from URSA patients and placental tissue of URSA mice displayed a marked reduction to NP group which is consistent with the original report [48]. Downregulation of *TGFB1* expression in trophoblast cells of URSA patients was further corroborated by scRNA‐seq analysis, and trophoblast cells communicated more actively with macrophages through the TGFB1‐TGFBR axis in NP group. Correlation analysis showed a positive relationship between NSUN2 and TGFB1 expression. In vitro experiments have shown that knocking down NSUN2 in trophoblast cells can inhibit the expression of TGF‐β1. Through m^5^C MeRIP‐seq we identified m^5^C modification sites on *TGFB1* mRNA. Further combining with the actinomycin‐D experiment, dual luciferase reporter gene detection, and m^5^C MeRIPqPCR, we established that NSUN2 enhances *TGFB1* mRNA stability in an m^5^C‐dependent manner. And in vitro methylation using purified NSUN2, demonstrated direct and site‐specific m^5^C deposition, but binding‐affinity measurements may help to further characterize the direct interaction between NSUN2 and *TGFB1* mRNA.

In addition to establishing NSUN2 as the m^5^C writer for *TGFB1* mRNA, we identified YBX1 as the cognate m^5^C reader that recognizes the modified transcript and preserves its stability. RNA pull‐down and YBX1‐RIP‐qPCR assays demonstrated that YBX1 specifically binds to m^5^C‐modified *TGFB1* 3’UTR in an NSUN2‐dependent manner, and YBX1 depletion markedly shortened *TGFB1* mRNA half‐life. These findings position YBX1 as an essential downstream effector of NSUN2‐mediated m^5^C modification, bridging epitranscriptomic regulation of trophoblast‐derived TGF‐β1 to macrophage polarization at the maternal‐fetal interface. Functionally, conditional knockout of NSUN2 in trophoblasts reduces TGF‐β1 secretion, relieving its active suppression of M1 polarization and thereby driving an M1 shift. This aligns with the established role of TGF‐β in restraining pro‐inflammatory programs [50, 51].

The immunoregulatory function of TGF‐β1 is predominantly mediated through the canonical Smad signaling pathway [52, 53]. Following receptor activation by TGF‐β1 binding, phosphorylated Smad2/3 forms a complex with Smad4. This Smad complex subsequently enters the nucleus, where it functions as a transcription regulator for downstream genes [54, 55]. In macrophages, activation of Smad2/3 directly promotes the transcription of M2 marker genes such as *YM1*, *IL‐10*, and *ARG‐1*, while suppressing the NF‐κB‐dependent expression of M1 pro‐inflammatory factors including *TNF‐α*, *IL‐1β*, and IL‐6, thereby polarizing macrophages toward an anti‐inflammatory, pro‐repair phenotype [50, 56]. Consistent with this pathway, we found that knockdown of NSUN2 in trophoblasts inhibited Smad2/3 phosphorylation in co‐cultured macrophages, whereas its overexpression had the opposite effect. Importantly, the decrease in Smad2/3 phosphorylation induced by NSUN2 knockdown could be rescued by supplementing the co‐culture system with exogenous TGF‐β1.

Finally, we overexpressed NSUN2 in URSA mouse models using adenovirus. The results showed that overexpression of NSUN2 in the placenta significantly reduced embryo resorption rate, upregulated TGFB1 expression in URSA placental tissue, and inhibited macrophage polarization toward M1 phenotype. These results indicate that the treatment strategy targeting NSUN2 has potential value in the clinical intervention of URSA. In summary, this study reveals the crucial role of the NSUN2‐TGFB1 axis in remolding the immune balance at MFI, offering a potential therapeutic strategy for URSA patients.

In conclusion, the present study establishes NSUN2 as a critical determinant of maternal‐fetal immune tolerance through m^5^C‐dependent stabilization of *TGFB1* mRNA in trophoblasts. NSUN2 deficiency leads to reduced TGF‐β1 expression, impaired Smad2/3 signaling in macrophages, and pathological M1 polarization, ultimately contributing to pregnancy loss. Conversely, NSUN2 restoration effectively rescues TGF‐β1 expression, rectifies macrophage imbalance, and alleviates embryo resorption in URSA models. These findings identify NSUN2 as a candidate diagnostic biomarker and promising therapeutic target for URSA, opening new avenues for clinical management of this challenging pregnancy complication.

## 4 Materials and Methods

### 4.1 Clinical Sample Collection

The study was approved by the Ethics Committee of Shandong Maternal and Child Health Hospital and registered in the International Traditional Medicine Clinical Trial Registry (ChiCTR1800018795). From September 2024 to August 2025, a total of 60 participants were enrolled, including 30 women with URSA and 30 women with NP. Chorionic villus samples were collected via suction aspiration during the procedure.

Exclusion criteria for the URSA group comprised: (1) genetic or chromosomal abnormalities, (2) endocrine disorders, (3) positive autoantibodies, (4) anatomical anomalies of the reproductive tract, and (5) any other known etiology of miscarriage. The inclusion criteria for the NP group are: (1) no history of spontaneous abortion or stillbirth, (2) no known conditions associated with pregnancy loss, (3) normal embryonic development with cardiac activity confirmed by ultrasound. All participants signed informed consent. Detailed clinical characteristics of both cohorts are available in Table S1.

### 4.2 Cell Culture and Intervention

HTR8/SVneo (HTR8), JEG‐3 cells and THP‐1 cell were sourced from Wuhan Procell Life Science & Technology Co., Ltd. HTR‐8 and THP‐1 cells were maintained in RPMI‐1640 medium (Bioind, Kibbuiz, Israel), and JEG‐3 cells were cultured in F12 medium (Bioind, Kibbuiz, Israel). All the cell culture medium contain 10% fetal bovine serum (Bioind, Kibbuiz, Israel) and 1% penicillin‐streptomycin.

The NSUN2 overexpression and knockdown lentiviral vectors were constructed by Genechem (Shanghai, China). After infecting cells with lentivirus, screening was performed using puromycin (53‐79‐2, MCE, USA) to obtain stable transfected cell lines. Target sequences of siRNAs, and lentivirus‐delivered shRNAs were provided in Table S2.

### 4.3 Transwell Migration Assay

Transwell chamber systems (24‐well plates, 3422, Costar) were adopted to evaluate migratory capacities of HTR‐8 and JEG‐3 cells. 1 × 10^5^ cells suspended in serum‐free medium were plated into upper compartments, with complete medium containing 10% FBS (04‐001‐1ACS, Bioind, Kibbuiz, Israel) in the lower chamber. After a 24 h incubation, invaded cells were fixed and stained with crystal violet, and representative images were captured under inverted microscopy.

### 4.4 CCK‐8 Assay

Cell viability was assessed using the CCK‐8 assay (Beyotime, C0037). HTR‐8 and JEG‐3 cells were seeded in 96‐well plates at a density of 5 × 10^3^ cells per well. After treatment, CCK‐8 reagent was added, and the cells were incubated at 37°C. Absorbance at 450 nm was measured using a microplate reader.

### 4.5 Apoptosis Assay

Apoptosis of HTR‐8 and JEG‐3 cells was detected using an Annexin‐V‐PE apoptosis kit (559763, BD Pharmingen, USA). Cells were collected and washed with cold PBS and incubated with PE‐Annexin‐V and 7‐AAD in binding buffer for 15 min in dark. The cell‐apoptosis proportion was analysed on a Beckman Coulter flow cytometer.

### 4.6 Isolation of Decidual Macrophage

Decidual tissues from NP women were digested with type IV collagenase (C2139, Sigma, USA) and DNase I (BS137, Biosharp, China) at 37°C with agitation at 200 rpm for 40 min. Single‐cell suspensions were subjected to Ficoll‐Paque density gradient centrifugation to obtain mononuclear cells, which were then stained with anti‐human CD45‐Alexa Fluor 700 (304023, Biolegend, USA) and anti‐human CD14‐FITC (367116, Biolegend, USA) antibodies. CD45^+^CD14^+^ macrophages were sorted by flow cytometry and cultured in RPMI 1640 medium containing 10% FBS.

### 4.7 Co‐Culture Model

To assess the impact of trophoblasts on macrophage polarization, a Transwell co‐culture system (Corning, USA) was employed. For the THP‐1 cell line model, THP‐1 monocytes were differentiated into M0 macrophages with 100 ng/mL PMA for 24 h. The M0 macrophages were then seeded into the lower chamber, and NSUN2‐knockdown or overexpressing HTR‐8 and JEG‐3 trophoblast cells were seeded into the upper chamber for 48 h of co‐culture. Isolated primary human CD45^+^CD14^+^ decidual macrophages were subjected to two culture conditions. For M1‐polarization analysis, macrophages were pre‐treated with 100 ng/mL LPS and 20 ng/mL IFN‐γ for 24 h. After medium renewal, cells were co‐cultured with NSUN2‐knockdown or NSUN2‐overexpressing HTR‐8 trophoblast cells via transwell inserts for another 48 h. For assessment of M2‐polarization, primary macrophages were directly incubated with trophoblasts in the transwell system for 48 h without prior stimulation. For TGF‐β1 supplementation, recombinant human TGF‐β1 (10 ng/mL, PeproTech, USA) was added to the co‐culture system for 48 h. After co‐culture, macrophages were collected for further analysis.

### 4.8 Mice

NSUN2‐floxed mice Wuhan Shulaibao Biotechnology Co., Ltd. (Manufacturing License No. SCXK (E) 2026‐0025) and Elf5‐Cre mice were obtained from Cyagen Biosciences Inc. (Manufacturing License No. SCXK (Su) 2022‐0016). Trophoblast‐specific *Nsun2* cKO mice were generated by crossing *Nsun2* ^fl/fl^ mice with *Elf5‐*Cre drivers. The *Elf5‐*Cre line drives specific Cre expression in the trophectoderm as early as E4.5, enabling targeted gene deletion in this lineage [57]. Genomic DNA for PCR genotyping was isolated from tail biopsies using an alkaline lysis protocol. Nsun2‐deficient placentas were identified by the genotype *Nsun2* ^fl/fl^ *Elf5‐*Cre, while *Nsun2* ^fl/fl^ placentas were used as controls. The Genotyping primer sequences were listed in Table S3.

Eight‐week‐old CBA/J and DBA/2 mice were sourced from Beijing Huafukang Biotechnology Co., Ltd. (Manufacturing License No. SCXK (Beijing) 2024‐0003). Female CBA/J mice were mated with male DBA/2 and BALB/c mice to construct the URSA and NP mouse models, respectively. We designated the day of vaginal plug observation as E0.5. On E7.5, URSA mice were administered NSUN2‐overexpressing adenovirus (Ad‐NSUN2) or control adenovirus (Ad‐NC) (Gene Chem, China) via tail vein injection. On E12.5, mice were euthanized and the embryo resorption rate was calculated. Fetal and placental weights were measured, and placental tissues were collected for further analysis. All animal experiments were performed in accordance with the “Code of Ethics and Welfare for Laboratory Animals” and were approved by the Ethics Committee of Shandong University of TCM (Approval No. SDUTCM20250624003).

### 4.9 4D Label Free Quantitative Proteomics

Villous tissues from URSA and NP subjects (n = 4 per group) were subjected to 4D label‐free quantitative proteomic analysis by Jingjie Biotechnology (Hangzhou, China). Samples were pulverized in liquid nitrogen and ultrasonically lysed in lysis buffer. After protein quality assessment, equal amounts of protein were enzymatically digested and analyzed by liquid chromatography‐tandem mass spectrometry.

Label‐free quantification intensities were log_2_‐transformed before statistical analysis. Proteins quantified in all eight samples were used for principal component analysis, and pairwise Pearson correlation coefficients were calculated to assess reproducibility among biological replicates. Proteins with valid quantitative ratios were compared between the URSA and NP groups. Differentially expressed proteins were defined by a fold change ≥ 1.5 or ≤ 0.67 and *P* < 0.05. Curated m^5^C‐ and m^6^A‐associated writers, erasers, and readers were matched to the quantified proteome, and their expression patterns were visualized using row‐scaled heatmaps and log_2_ fold changes.

### 4.10 RNA‐Seq

RNA‑seq was performed on NSUN2‑depleted and control JEG‑3 cells (n = 3) by CloudSeq Inc. Total RNA was enriched for mRNA, fragmented to ∼300 nt, and reverse‑transcribed with random hexamers and dUTP was incorporated during second‑strand synthesis to preserve strand specificity. UMI‑containing adapters were ligated for precise duplicate removal, and libraries were sequenced on a paired‑end 150 bp platform. Raw reads were processed with fastp (adapter trimming, low‑quality filtering, UMI extraction), aligned to HG38 using HISAT2 (v2.2.1), and quantified at the gene level with featureCounts followed by UMI‑tools deduplication. Differential expression analysis was performed using edgeR with thresholds of |log_2_FC| ≥ 1.0 and *P* < 0.05. The complete differentially expressed gene table (including gene identifiers, symbols, counts, FPKM, CPM, fold change, log_2_FC, *P* value, FDR, and annotation) is provided in the supporting information. Functional enrichment analyses were performed using clusterProfiler, including GO/KEGG over‑representation analysis (with all detected genes as background) and GSEA (ranked by log_2_FC). *P* values were adjusted by the Benjamini‑Hochberg method, with adjusted *P* < 0.05 considered significant. NES and corresponding FDRs are reported in the supporting information.

### 4.11 m^5^C MeRIP‐Seq

m^5^C MeRIP‐seq of NSUN2‐depleted and control JEG‐3 cells (n = 3) was performed by CloudSeq Inc. Total RNA was fragmented to ∼200 nt and incubated with m^5^C antibody‐coated magnetic beads at 4°C for 4 h. After elution and purification, both IP and input RNA were subjected to library construction using the GenSeq Low Input Whole RNA Library Prep Kit and sequenced on an MGI T7 platform (PE). Raw reads were trimmed with cutadapt (v1.9.3) and aligned to HG38 using HISAT2 (v2.0.4). m^5^C peaks were called with MACS (v1.4.2) by comparing IP versus input libraries, and differentially methylated peaks between NSUN2‐depleted and control groups were identified using diffReps (v1.55.6), retaining only exonic peaks within mRNA biotypes. Differentially methylated peaks were defined as fold change ≥ 2 and *P* < 0.001. Complete peak and differential peak tables, including genomic coordinates, peak length, transcript ID, gene symbol, peak enrichment, fold change, *P* value, FDR, regulation direction, and gene annotation are provided in the supporting information.

### 4.12 Single‐Cell RNA‐Seq (ScRNA‐seq) Processing and Annotation

Public scRNA‐seq data from human decidual and villous samples (URSA and NP groups) were obtained from the Sequence Read Archive (accession SRP400574) [58]. FASTQ files were processed with STARsolo against the GRCh38‐2020‐A reference. The 10x Genomics v3 barcode structure was specified as a 16‐nt cell barcode followed by a 12‐nt unique molecular identifier, the 3M‐february‐2018 barcode whitelist was used, droplets were called with EmptyDrops_CR, and Gene and Velocyto count matrices were generated. The filtered gene‐count matrices were imported into Seurat and merged while retaining sample identity. The initial merged matrix contained 139 138 cells and 36 601 genes. Genes detected in fewer than 20 cells were removed. After sample‐wise inspection and filtering of outlying nCount_RNA, nFeature_RNA, and mitochondrial‐read distributions, the following fixed cell‐level criteria were applied: nFeature_RNA ≥ 500, nCount_RNA ≥ 1,000, mitochondrial transcripts ≤ 15%, and hemoglobin transcripts ≤ 1%. This procedure retained 86 198 cells and 25 015 genes.

Counts were normalized with Seurat LogNormalize using a scale factor of 10 000. The 2000 most variable genes were selected with the variance‐stabilizing transformation method, and 30 principal components were calculated. Samples were integrated with scVI [59].The t‐SNE embedding, shared nearest neighbor (SNN) graph, and clustering were computed based on the first 30 dimensions of the integrated scVI latent space. Clustering resolutions from 0.1 to 2.0 in increments of 0.1 were evaluated with clustree; resolution 0.8 was selected on the basis of cluster stability and marker coherence. Cell types were annotated by canonical markers. Related subtypes were merged into major cell classes. Major classes containing more than 500 cells were retained for the final overview analyses, yielding 82 246 cells and 25 015 genes.

### 4.13 Single‐Cell RNA Modification Signature Scoring

Per‐cell RNA‐modification signature scores were calculated using UCell [60], a rank‐based method based on the Mann‐Whitney U statistic that is comparatively robust to differences in library size and data sparsity. Curated gene sets for each RNA‐modification pathway were intersected with genes detected in the scRNA‐seq matrix, and signatures containing at least two detected genes were scored using AddModuleScore_UCell. The C‐to‐U editing signature was excluded because only one signature gene was detected. The m^5^C signature comprised NOP2, NSUN2, NSUN3, NSUN4, NSUN5, NSUN6, NSUN7, TRDMT1, DNMT1, DNMT3A, DNMT3B, ALYREF, YBX1, TET1, TET2, and TET3. The complete gene sets used for all RNA‐modification signatures are provided in Table S4. NP and URSA per‐cell scores were compared using two‐sided Wilcoxon rank‐sum tests. Nominal *P* < 0.05 was considered significant for these exploratory signature‐score comparisons.

### 4.14 Cell‐Cell Communication Analysis

Cell‐cell communication was inferred using CellChat [61] from normalized gene‐expression data and the curated CellChatDB ligand‐receptor database. All ligand‐receptor pairs in the trophoblast‐to‐macrophage and macrophage‐to‐trophoblast directions were extracted separately for NP and URSA samples. Pairs with CellChat permutation *P* < 0.05 and Benjamini‐Hochberg‐adjusted route‐level FDR < 0.05 were considered significant. Significant pairs were ranked by communication probability within each directed route and visualized using pathway heatmaps, ligand‐receptor bubble plots, and bidirectional network diagrams. The relative rank and condition specificity of TGFB‐TGFBR interactions were assessed against the complete set of significant pairs. For NSUN2‐stratified analysis, normalized NSUN2 expression was extracted from the Seurat RNA assay. Trophoblasts with expression > 0 were classified as NSUN2‐positive (NSUN2^+^) and those with expression = 0 as NSUN2‐negative (NSUN2^−^). The NP dataset contained 2422 NSUN2‐positive 12 246 NSUN2‐negative trophoblasts; the URSA dataset contained 449 and 7281 cells, respectively. CellChat was rerun after replacing the trophoblast label with these two strata while retaining all other major cell‐type labels. Decidual macrophage subclusters were described as M1‐like and M2‐like, rather than as fixed binary states, because macrophage activation occurs along a continuum. M1‐like annotation was supported by IL1B, TNF, CXCL8, CCL3, CCL4, NFKBIA, FCGR3A, and LST1, whereas M2‐like annotation was supported by CD163, MRC1, MSR1, MERTK, C1QA, C1QB, C1QC, APOE, FOLR2, and IL10.

### 4.15 qRT‐PCR

Total RNA of cells and tissue were obtained using Trizol. RNA was reverse transcribed into cDNA using the PrimeScript RT kit (Toyobo, Osaka, Japan). PCR amplification was carried out by SYBR Green (CW2601, cwbio, China). The data were calculated using the 2^−ΔΔCt^ method with all primer sequences detailed in Table S5.

### 4.16 Western Blotting (WB)

Total proteins from cells or tissues were extracted using RIPA lysis buffer containing protein phosphatase inhibitor and protease inhibitor. The sample were separated by SDS polyacrylamide gels. The following antibodies were used: NSUN2 (20854‐1‐AP, Proteintech, China), TGF‐β1 (ab215715, Abcam, USA), iNOS (ab178945, abcam, USA), ARG‐1 (ab124917, abcam, USA), β‐actin (ab7817, abcam, USA), GAPDH (ab181603, abcam, USA), p‐Smad2/3 (F2846, Selleck, China), Smad2/3 (ab202445, abcam, USA).

### 4.17 Immunohistochemistry (IHC)

Tissues were fixed with 4% paraformaldehyde. For detection, a universal Two‐Step Detection Kit (PV‐9000, Zhongshan Jinqiao, China) was applied as per the protocol provided with the kit. Paraffin sections were baked at 60°C for 2 h, dewaxed, and rehydrated. After antigen repair and inhibition of endogenous peroxidase activity, the sections were incubated with NSUN2 primary antibody (20854‐1‐AP, Proteintech, China) overnight at 4°C. Finally, DAB chromogen was applied for 8 min for color development. IHC quantification was performed by ImageJ. Relative expression was determined by normalizing each sample's raw % area to the mean % area of the NP cohort.

### 4.18 Immunofluorescence (IF)

Human villous tissues were fixed with 4 % paraformaldehyde, embedded in paraffin and sliced into 5 µm sections. After dewaxing and antigen retrieval, sections were blocked and incubated with anti‐m^5^C antibody (ab214727, abcam, USA) overnight at 4 °C. After incubation with Goat Anti‐Rabbit IgG H&L (Alexa FluorR 647) antibody (ab150157, abcam, USA) and DAPI staining. Image‐J software was applied to calculate m^5^C fluorescence intensity within trophoblasts.

### 4.19 Flow Cytometry

For cells: After sample treatment, the single cells were stained with PE anti‐human CD86 (12‐0869‐42, Invitrogen, USA), FITC anti‐human CD11b (53‐0112‐82, Invitrogen, USA), APC anti‐human CD206 (17‐2069‐42, Invitrogen, USA) at 4°C for 30 min.

For tissues: Mouse decidual tissue was washed with ice‐cold PBS and minced into 1–3 mm^3^ fragments. The fragments were then digested in RPMI 1640 medium containing type IV collagenase (C2139, Sigma, USA) and DNase I (BS137, Biosharp, China) at 37°C with agitation at 200 rpm for 40 min. After filtration through a 100 µm cell filter, the cell suspension was subjected to Percoll gradient centrifugation to isolate the mononuclear cells. The following antibodies were used for flow cytometry: APC anti‐mouse F4/80 (17‐4801‐82, Invitrogen, USA), PE anti‐mouse CD80 (104707, Biolegend, USA), PE anti‐mouse CD206 (141706, Biolegend, USA), Alexa Fluor 700 anti‐mouse CD45 (147716, Biolegend, USA), APC anti‐mouse CD8a (100711, Biolegend, USA), Brilliant Violet 421 anti‐mouse CD4 (100443, Biolegend, USA), Alexa Fluor 488 anti‐mouse CD3 (100210, Biolegend, USA), FITC anti‐mouse CD80 (104705, Biolegend, USA), APC anti‐mouse NK1.1 (17‐5941‐82, Invitrogen) at 4°C for 30 min.

### 4.20 ELISA

The concentrations of IL‐10, TGF‑β1, and TNF‑α in the co‐culture supernatants was performed by specific ELISA kits (Human IL‐10: EK110, Human/Mouse/Rat TGF‐β1: EK98, Human TNF‐α: EK182) according to the manufacturers’ instructions. Human villous tissues from NP and URSA patients were fully homogenized in cold PBS supplemented with 1 mM Phenylmethylsulfonyl‐fluoride (PMSF, FJR0010, Solarbio, China), followed by centrifugation. The supernatants were collected, and TGF‐β1 concentrations were examined using a commercial human TGF‐β1 ELISA kit (EK98) in accordance with the manufacturer's instructions. The All the ELISA kits come from MULTI SCIENCES (China).

### 4.21 RNA m^5^C Dot Blot Assay

Total RNA was heated at 95°C for 3 min to induce denaturation, and then spotted onto a nitrocellulose membrane. The membrane was fixed at 37°C for 30 min, stained with 0.02% methylene blue (61‐73‐4, MCE, USA) to verify RNA loading. Anti‐m^5^C antibody (ab214727, abcam, USA) was incubated overnight at 4°C.

### 4.22 mRNA Stability Assay

To assess mRNA stability, HTR8 and JEG‐3 cells were treated with 5 µg/mL actinomycin D (50‐76‐0, Sigma, USA). RNA was harvested at 0, 2, 4, and 6 h using TRIzol, reverse‐transcribed (PrimeScript RT kit, Toyobo, Japan), and analyzed via qRT‐PCR. Relative mRNA levels were calculated by normalization to the 0 h sample.

### 4.23 RIP Assay

RIP was performed with a commercial kit (P002, Geneseed Biotech, China) according to the manufacturer's protocol. Briefly, cell lysates were incubated with magnetic beads conjugated either with an anti‐NSUN2 antibody or with a normal IgG control. Following immunoprecipitation, the co‐precipitated RNAs were extracted and purified, and their enrichment of specific target mRNAs was quantified by qRT‐PCR.

### 4.24 m^5^C MeRIP‐qPCR

The methylated RIP was performed using m^5^C MeRIP Kit (GS‐ET‐003, Cloudseq Biotech Inc, China) in accordance with the protocol. Approximately 18 µg of total RNA was isolated from transfected HTR8 and JEG‐3 cells and chemically fragmented. Fragmented RNA was incubated with magnetic beads pre‐coupled with m^5^C antibody or an isotype control IgG. After washing and elution, the co‐precipitated RNA was purified and analyzed via qRT‐PCR. Primer sequences were listed in Table S5.

### 4.25 Dual‐Luciferase Reporter Assays

Luciferase reporter plasmids containing *TGFB1* mRNA 3’UTR wild type (WT) and two single site mutants (Mut 1 carried a C‐to‐T substitution in the CCTGGGG motif, Mut 2 carried a C‐to‐T substitution in the ACTGCGG motif) and Mut 3 contained both single‐site substitutions simultaneously were synthesized by Genechem (Shanghai, China). HET293T cells were co‐transfected with NSUN2 siRNA or negative control (si‐NC) and WT or Mut luciferase reporter plasmid. Luciferase reporter activity was anlysised by the Dual‐Luciferase Reporter Assay System (E1910, Promega, USA) using a GloMax 20/20 Luminometer (Promega, USA).

### 4.26 In Vitro m^5^C Modification Assay

The plasmid containing *TGFB1* mRNA 3’UTR and then digested with restriction enzymes to produce linear DNA, which was purified with Universal DNA Purification Kit (DP24‐02, TIANGEN). Then in vitro RNA transcription was carried out by HyperScribe T7 High‐Yield RNA Synthesis Kit (K047, APEXBIO, USA). Purified transcribed RNA was subjected to in vitro m^5^C modification reaction. The 100 µL reaction system contained 2 µm recombinant human NSUN2 protein (CSB‐EP626626HU, Cusabio, China), 1 µm RNA substrate, 10 µL of 10× methylation buffer and 80 µm S‐adenosylmethionine (SAM) (A4377‐5MG, Sigma–Aldrich, USA). Nuclease free water was supplemented to adjust the total volume to 100 µL. After incubation at 37 °C for 4 h, the reaction was terminated by heating at 95°C for 2 min. Finally, RNA was purified and subjected to dot blot analysis.

### 4.27 RNA‐Pull‐Down Assay

Biotin‐labeled *TGFB1* [C] (without m^5^C) and *TGFB1* [m^5^C] (m^5^C‐modified) RNA fragments at the predicted m^5^C site were synthesized in vitro (as detailed in Table S2) and then incubated with protein extracts from JEG‐3 cells following the manufacturer's protocols of the RNA‐protein pull‐down Kit (P811S, Beyotime, China). After thorough washing, the bead‐bound proteins were eluted, separated by SDS‐PAGE, and the enrichment of YBX1 (ab76149, abcam, USA) and ALYREF(ab202894, abcam, USA) was detected via western blotting analysis.

### 4.28 Statistical Analysis

Statistical analysis was performed with GraphPad Prism 7.0. Data are expressed as mean ± SEM. Differences between two groups were assessed by two‐tailed Student's t‐test. For comparisons, two‐tailed Student's t‐test (two groups) or two‐way ANOVA with Tukey's post hoc test (multiple groups) was applied. Linear correlations were analyzed by Pearson's correlation coefficient (r). *P*< 0.05 was considered statistically significant.

## Author Contributions

X.Z., X.L. and Z.Z. conceived the study and acquired funding. X.Z performed the molecular biology experiments and drafted the original manuscript. X.L. conducted the bioinformatics analysis. X.Y., Q.H. and F.S. carried out the animal studies. W.S., L.L. and C.C. performed the in vitro functional assays. X.L. and C.S. were responsible for clinical sample collection. All the authors reviewed, edited, and approved the final version of the manuscript.

## Funding

This study was assisted by National Natural Science Foundation of China (82274575, 82474564, 82505658, and 82471704), the Major Basic Research Project of Shandong Natural Science Foundation (ZR2023ZD56), the Science & Technology Cooperation Program of Shandong (2025KJHZ035), the Natural Science Foundation of Shandong Province (ZR2022LZY011, ZR2023QC167), the Co‐construction project of the State Administration of TCM (GZY‐KJS‐SD‐2023‐034 and GZY‐KJS‐SD‐2023‐046), the Shandong Province Taishan Scholar Project (No. tstp20240513), the Central Government Guides Local Science and Technology Development Fund Projects of Shandong Province (YDZX20203700001407), the Key Laboratory of TCM Classical Theory, the Ministry of Education, the Open Research Project, the National Youth Qihuang Scholar Training Program and the Shandong Province TCM High Level Talent Cultivation Project, the Undergraduate Education Reform Project of Shandong Province (Z2024231), Shandong University of Traditional Chinese Medicine Bianque Scholar Climbing Program‐Li Xia, 2026 Excellence Projects of the China‐Africa Universities 100 Cooperation Plan.

## Conflicts of Interest

The authors declare no conflicts of interest.

## Supporting information




**Supporting File 1**: advs77562‐sup‐0001‐SuppMat.docx.

**Supporting File 2**: advs77562‐sup‐0002‐SuppMat.xlsx.

## Data Availability

The data that support the findings of this study are available from the corresponding author upon reasonable request.
