# Supplementary material for: NSUN2‐Mediated m5C Modification of TGFB1 in Trophoblasts Remodels Macrophage Function to Prevent URSA
Source: Adv Sci (Weinh). 2026 Sep 13:e77562. Online ahead of print. doi: 10.1002/advs.77562 (PMC13572827; doi:10.1002/advs.77562)
Supplement: Supplementary file 1 [file ADVS-9999-e77562-s002.docx]

Supporting Information

**NSUN2-mediated m^5^C modification of *TGFB1* in trophoblasts remodels macrophage function to prevent URSA**

Xiaoxiao Zhu^1,2†^, Xinkui Liu^1,2†^, Xiaoyan Yu^1,2^, Qiaoqiao Han^1,2^, Feifei Shi^1,2^, Weiwei Song^3^, Lu Liu^1,2^, Chu Chu^1,2^, Chenyang Shen^1,2^, Zhen Zhang^1,2^*, Xia Li^1,2,4^*

**This file includes**:

1. Supplementary Figures 1 to 4
2. Supplementary Tables 1 to 5

**Figure S1. Integrative proteomic and functional analysis reveals NSUN2 deficiency drives embryonic resorption in URSA.**

(**A**) Dot plot showing the expression of representative canonical marker genes across the nine annotated major cell populations in the combined NP and URSA dataset. Dot size represents the percentage of cells within each population with normalized expression greater than zero, and color represents the gene-wise scaled average expression calculated from the log-normalized RNA assay. (**B**) Principal component analysis of 3,639 proteins quantified in all samples (n = 4 per group). (**C**) Pearson correlation analysis based on log_2_-transformed LFQ intensities. (**D**) Heatmap showing the relative abundance of detected RNA modification-associated proteins. Values represent row-scaled log_2_ LFQ intensities. (**E**) Log_2_ fold changes of m^5^C- and m^6^A-associated proteins in URSA versus NP tissues. Blue bars indicate significantly downregulated proteins, whereas gray bars indicate quantified proteins that did not meet the differential-expression criteria. Dashed lines indicate the fold-change thresholds. (**F**) Genotype analysis of the offspring from female *Nsun2*^fl/fl^ mice crossed with male *Nsun2*^fl/+^*Elf5-*Cre mice. (**G)** NSUN2 protein level in E12.5 *Nsun2*^fl/fl^ and *Nsun2*^fl/fl^*Elf5-*Cre mice placental tissues (n = 3). (**H)** Genotyping of the mouse placentas for the *Nsun2* conditional allele and Cre recombinase. The conditional *Nsun2* allele shows bands at 301 bp (WT), 355 bp (Homozygous, HO), or both (HET). Cre status is determined by the presence (Cre^+^) or absence (Cre^-^) of a 362 bp band. Error bars represent means ± SEM. ***P* < 0.01.

**Figure S2. NSUN2 does not significantly affect proliferation, migration, or apoptosis of trophoblast cells.**

(**A**, **B**) NSUN2 mRNA levels in HTR8/ JEG-3 NSUN2 knockdown and overexpression stable cell lines. (**C, D**) WB showing the expression levels of NSUN2 in NSUN2 knockdown or overexpression HTR8 and JEG-3 stable cell lines. (**E, F**) CCK-8 analysis for cell proliferation following NSUN2 knockdown or overexpression. (**G, H**) Flow cytometry was used to detect the apoptotic proportion of trophoblast cells. (**I, J**) Transwell-migration assays evaluated cell migratory potential. **All data are representative of three independent experiments.** Error bars represent means ± SEM. ns: no statistical difference, **P* < 0.05, ***P* < 0.01, ****P* < 0.001, *****P* < 0.0001.

**Figure S3. NSUN2 deficiency in trophoblasts promotes M1-like macrophage polarization.**

(**A)** Volcano plot illustrating differentially expressed genes in NSUN2 knockdown JEG-3 stable cell lines based on RNA-seq data (n = 3). (**B)** Single-cell communication analysis of the differences in communication strength between NSUN2^-^ and NSUN2^+^ trophoblast cells. (**C**) Violin plots showing macrophage-directed CellChat communication probabilities from NSUN2^-^ and NSUN2^+^ trophoblasts in NP samples. Boxes indicate the median and interquartile range. (**D**) Gating strategy for flow cytometric analysis of mouse decidual immune cell subsets. (**E**) Schematic diagram of the co-culture system comprising THP-1-derived macrophages or primary human decidual macrophages with stable HTR-8 and JEG-3 trophoblast cells line with NSUN2 knockdown or overexpression. (**F, G**) The proportion of M1 (CD11b^+^ CD86^+^) and M2 (CD11b^+^CD206^+^) in THP-1drived macrophages co-cultured with JEG-3. (**H**, **I)** qRT-PCR detection of *NOS2*, *CD86*, *TNF-α*, *ARG-1* and *IL-10* mRNA level in THP-1 co-cultured with HTR8 and JEG-3. (**J**, **K)** WB showing the protein levels of iNOS and ARG-1in THP-1drived macrophages co-cultured with JEG-3. (**L**, **M)** ELISA analysis the level of TNF-α and IL-10 in the supernatant of co-culture system with JEG-3. (**N-Q**) For M1-phenotype detection, primary human CD45^+^CD14^+^ decidual macrophages were stimulated with LPS plus IFN-γ for 24 h, then the culture medium was replaced before co-culture with trophoblasts in transwell chambers. For M2-related analysis, primary macrophages were directly co-cultured with trophoblasts. (**N, O**) mRNA levels of M1-associated genes (*NOS2*, *TNF-α*) and M2-associated genes (*IL-10*, *ARG-1*) were measured by qRT-PCR in primary macrophages after co-culture with NSUN2 knockdown or overexpression HTR8 cells. (**P, Q**) WB detection of iNOS and ARG-1 in primary macrophages after co-culture with NSUN2 knockdown or overexpression HTR8 cells. NSUN2^+^, NSUN2-positive; NSUN2^-^, NSUN2-negative. **Data in** F-Q **are representative of three independent experiments.** Error bars represent means ± SEM. ns: no statistical difference, **P* < 0.05, ***P* < 0.01, ****P* < 0.001, *****P* < 0.0001.

**Figure S4. The pathological relevance of TGF-β1 and its potential inflence on cell communications in URSA**

(**A**) Heatmap for the enrichment scores of hallmark gene sets in sh-NC vs. sh-NSUN2 group. (**B**) Differences in enrichment scores of TGF-beta signaling pathway between the sh-NC and sh-NSUN2 groups. (**C**, **D**) *TGFB1* mRNA (n = 10) and protein levels (n = 6) in placenta tissues of NP and URSA mice. (**E**) The secreted TGF-β1 concentration in human chorionic villus tissue supernatants from NP and URSA patients were quantified by ELISA (n = 15). (**F**) ROC curve evaluating the diagnostic value of TGFB1 in villous tissues (n = 30). (**G**) Heatmap of significant signaling pathways. Tile color indicates summed communication probability, and numbers indicate significant ligand-receptor pair counts. (**H**) Bubble plot of the top significant ligand-receptor pairs in each communication direction, with significant TGFB-TGFBR pairs retained. Bubble size represents communication probability, and color indicates route-level FDR. (**I**) Bidirectional communication networks between trophoblasts and macrophages. Edge width represents summed probability, and labels indicate ligand-receptor pair counts, total probability, and TGFB-TGFBR contribution. (**J**) Relative ranks and probabilities of TGFB-TGFBR interactions in NP, along with their proportional contribution to total significant communication in NP and URSA. Significant interactions were defined by CellChat with permutation *P* < 0.05 and BH‑adjusted FDR < 0.05. Error bars represent means ± SEM. ***P* < 0.01, ****P* < 0.001, *****P* < 0.0001.

**Table S1**. **Clinical characteristics of patients.**

| **Patients** | **URSA** | **NP** | ***P*** |
| --- | --- | --- | --- |
|  | **(mean ± SEM, n = 30)** | **(mean ± SEM, n = 30)** |  |
| Age (years) | 33.0667 ± 0.9579 | 31.3 ± 1.0731 | 0.2321 |
| Number of miscarriages | 2.6333 ± 0.1200 | 0 | < 0.0001 |
| Gestation age (weeks) | 7.5667 ± 0.2435 | 7.7333 ± 0.2941 | 0.6694 |

**Table S2. Target sequences of siRNAs, lentivirus-delivered shRNAs and TGFB1 RNA-probe sequences used in this study.**

| **shRNA** | **Sequence (5’-3’)** |
| --- | --- |
| si-NC | TTCTCCGAACGUGUCACGT |
| si-YBX1#1 | GGACTTTCAAGTCTCCCGATGA |
| si-YBX1#2 | CAACCAGGGTGCAGGAGAATT |
| LV-sh NC | TTCTCCGAACGTGTCACGT |
| LV- sh NSUN2#1 | CCCAAGAATGAACGGCTTCAT |
| LV- sh NSUN2#2 | GAGCGATGCCTTAGGATATTA |
| **Probe sequences for RNA pulldown** | |
| TGFB1 [C] | AGCCCACCUGGGGCCCCAUUAAAGAUGGAGAGAGGACUGCGGAUCUCU-Biotin |
| TGFB1 [m^5^C] | AGCCCAC[m^5^C]UGGGGCCCCAUUAAAGAUGGAGAGAGGA[m^5^C]UGCGGAUCUCUGU-Biotin |

**Table S3. Primers used for PCR amplification and PCR genotyping of the *Nsun2*^flox/flox^ and *Elf5*-Cre transgenes.**

| **Primers** | **Sequences (5’-3’)** |
| --- | --- |
| *Elf5*-F  *Elf5*-R | CCTGAGAGAGCCAGGAGTGTTTA  ACATGTCCATCAGGTTCTTGCG |
| *Nsun2*-mutant  *Nsun2*-WT | AACCTGATGGCTTGCCTGTG  ACATTTGACCTCAAACCGCC |

**Table S4. RNA modification gene panel used in this study.**

| **Modification** | **Genes** |
| --- | --- |
| m^6^A | METTL3, METTL14, WTAP, RBM15, RBM15B, ZC3H13, VIRMA, CBLL1, YTHDF1, YTHDF2, YTHDF3, YTHDC1, YTHDC2, IGF2BP1, IGF2BP2, IGF2BP3, HNRNPA2B1, HNRNPC, FMR1, LRPPRC, FTO, ALKBH5 |
| m^5^C | NOP2, NSUN2, NSUN3, NSUN4, NSUN5, NSUN6, NSUN7, TRDMT1, DNMT1, DNMT3A, DNMT3B, ALYREF, YBX1, TET1, TET2, TET3 |
| m^1^A | TRMT6, TRMT61A, TRMT61B, TRMT10C, YTHDF1, YTHDF2, YTHDF3, YTHDC1, ALKBH1, ALKBH3 |
| m^7^G | METTL1, WDR4, RNMT, RAMAC, TGS1, NCBP1, NCBP2, NCBP3, EIF4E, EIF4E2, EIF4E3, GEMIN5 |
| A-to-I RNA Editing  (A-to-I) | ADAR, ADARB1, ADARB2 |
| Pseudouridylation (Ψ) | PUS1, PUS3, PUS7, PUS7L, TRUB1, TRUB2, DKC1, RPUSD1, RPUSD2, RPUSD3, RPUSD4 |
| 2'-O-methylation  (2'-O-Me) | FBL, CMTR1, CMTR2, MRM1, MRM2, MRM3, FTSJ3 |
| ac^4^C | NAT10, THUMPD1 |
| m^6^Am | PCIF1, FTO |
| m^3^C | METTL2A, METTL2B, METTL6, METTL8 |

**Table S5. List of primers used in the study.**

| **Gene** | **Forward primer (5'-3')** | **Reverse primer (5'-3')** |
| --- | --- | --- |
| **Homo sapiens** |  |  |
| *NSUN2* | ACAGCCACTGAGTTGGTATCC | TTATGATGAGGCCGCACGTT |
| *TGFB1* | TGGTGGAAACCCACAACGAA | GAGCAACACGGGTTCAGGTA |
| *NOS2* | AGCCCTCAGAGTACAGCAAGTG | AGCAGGAAGCCAGCAGACAC |
| *ARG-1* | TGGACAGACTAGGAATTGGCA | CCAGTCCGTCAACATCAAAACT |
| *CD86* | GCTGTAATCCAAGGAATGTGGTC | CCATCAGCTTGTCTGTTTCATTCC |
| *IL-10* | TCTCCGAGATGCCTTCAGCAGA | TCAGACAAGGCTTGGCAACCCA |
| *TNF-*α | TCTCGAACCCCGAGTGACAA | TGAAGAGGACCTGGGAGTAG |
| *GAPDH* | CACCCACTCCTCCACCTTTGAC | GTCCACCACCCTGTTGCTGTAG |
| **Mus musculus** |  |  |
| *Tgfb1* | AGGACCTGGGTTGGAAGTGG | GCCCGGGTTGTGTTGGTT |
| *Gapdh* | TGTCTCCTGCGACTTCAACA | GGTGGTCCAGGGTTTCTTACT |
| *Nsun2* | TACCATGTTCCCACCAACGG | ACGTTTGTTCCACGGCATTG |
| **Primers for m^5^C MeRIP-qPCR** | |  |
| *TGFB1* ACAGAGATCCGCAGTCCTC | | GCTCCCTTGCCCATGGGG |
